# Limits on selecting multiple items from working memory: the role of context and item competition

**DOI:** 10.3389/fcogn.2025.1668316

**Published:** 2025-11-11

**Authors:** Chen Tiferet-Dweck, Steven Zamora-Romero, Kerstin Unger

**Affiliations:** 1 Department of Psychology, Queens College, City University of New York, New York, NY, United States; 2 The Graduate Center, City University of New York, New York, NY, United States

**Keywords:** working memory, multi-item access, retrocuing, output gating, working memory selection, context competition, item competition, dual-access

## Abstract

To support goal-directed behavior, working memory (WM) must allow flexible and efficient access to its contents. While much research has examined how individual items are selected from WM, less is known about the principles guiding the simultaneous selection of multiple contents. Prior work has shown that multi-item access is slower and more error-prone than single-item access. The present study aims to clarify the mechanisms underlying this cost. Specifically, we examine whether the performance decline identified in our prior work reflects increased competition among memory items, among the context representations that guide selection, or both. To distinguish between these possibilities, we used a spatial retrocuing paradigm in which we independently manipulated (1) the number of cued locations (i.e., spatial contexts) and (2) the number of to-be-selected memory items. Across five experiments, we consistently found that cueing two spatial locations—compared to one—substantially delayed selection, even when the number of relevant items was held constant. By contrast, relevant set size had a smaller and less consistent effect on selection speed. These results suggest that the bottleneck in multi-item WM selection arises from the need to use multiple contexts to retrieve the associated memory contents.

## Introduction

Working memory (WM) is a highly capacity-limited system that enables us to keep information in a directly accessible state to support ongoing behavior. Since task demands often evolve over time or change with the prevailing context, not all of the information we maintain in WM is behaviorally relevant at any given moment. To act effectively, we need to be able to dynamically control which subset of WM contents will become available to attentional or motor systems (Buschmann and Miller, 2022; Chatham and Badre, 2015; Myers, 2022). Consider, for example, a student who is taking a standardized test such as the GRE or SAT. After reading a complex passage about environmental regulation, he may be asked to summarize the author's main argument regarding a rule on carbon taxes. Prior to this specific query, he likely retained a broad range of details in WM—which may include renewable energy targets, rules for climate risk disclosures, regulatory bans, and more. Once it becomes clear that only the discussion of carbon taxes is relevant, he must prioritize the subset of information that relates specifically to that topic.

Several neurocomputational WM models suggest that such prioritization relies on temporary^1^ bindings between memory contents and contextual representations, e.g., an item's spatial location or its position in a sequence (Lewandowsky and Farrell, 2008; Manohar et al., 2019; Oberauer and Lin, 2017, 2023; Swan and Wyble, 2014). Much of the empirical work to date has focused on how these item-context bindings constrain the selection of *individual* WM elements. However, many real-world tasks require accessing *multiple* WM items simultaneously. So far, comparatively little is known about the principles governing multi-item retrieval. We have previously shown that selecting multiple items from WM is associated with increased reaction times and higher error rates (Tiferet-Dweck et al., 2025; see also Oberauer, 2001, 2005). The present study aims to clarify the mechanisms underlying this cost. Specifically, we examine whether the performance decline identified in our prior work reflects increased competition among memory items, among the context representations that guide selection, or both.

### Mechanisms of selective working memory access

Many theories of WM share the assumption that memory contents are accessed through cue-based retrieval. A prominent example is the interference model (Oberauer and Lin, 2017, 2023), which posits that WM encoding involves the formation of temporary bindings between memory items (such as visual objects or words) and contextual features. Context, here, is functionally defined and refers to any information that can be used to identify the maintained WM contents. This may include sensory and semantic features of the attended items themselves, or more peripheral aspects, such as spatiotemporal information. During retrieval, presentation of a context cue reactivates the item that was bound to the corresponding context at encoding. This boost of activation is thought to move the item into a privileged state, referred to as the focus of attention, that allows it to directly influence cognitive processes and behavior (Cowan, 2001; Oberauer et al., 2013). According to the interference model, WM access is constrained by the strength and precision of the item-context bindings. Since both items and contexts are represented with limited precision, a cued context may activate not only the target item but also non-target items that they share similar, and therefore overlapping, contexts or features. Such representational overlap increases competition among retrieval candidates and produces mutual interference (distortion), which can manifest as slower, less efficient, or even failed retrieval, as well as confusion errors. The likelihood of interference increases with memory set size: the more items are maintained, the greater the chance that their representations—or the associated context representations—overlap. In addition, higher WM load is thought to result in a general decline in precision and encoding strength, possibly as a consequence of divisive normalization when multiple items are represented in the same neural population (Buschman, 2021; Busse et al., 2009; Carandini and Heeger, 2011). Together, these mechanisms account for the well-established set size effect, namely that both response speed and accuracy of WM retrieval decline as the number of maintained items increases.

At their core, the interference model and related theories construe WM access as a stimulus-driven, bottom-up process. As such, they cannot fully account for the significant flexibility and level of control we have about what we maintain in WM, or when and how we use its contents. An exclusively stimulus-driven access to WM may not be sufficient in the face of cue ambiguity and representational overlap, competing long-term memory influences, or the need to act upon abstract internal states rather than concrete external features. Accordingly, there is little doubt that both WM encoding and access are under goal-directed, executive control (Buschmann and Miller, 2022; Chatham and Badre, 2015).

One class of potential mechanisms underlying top-down control of WM contents has been described by the WM gating framework (Chatham and Badre, 2015; Frank and Badre, 2012). According to this theory, access to WM is controlled by interactions between prefrontal cortex (PFC) and basal ganglia. More precisely, the model posits that the PFC actively maintains memory representations—possibly in concert with sensory areas—while the basal ganglia act as a gate to regulate information flow within WM and between WM and motor/attentional systems. The passage of information is hierarchically organized such that some prefrontal representations serve as higher-order context by determining which among the several cognitive or motor representations at the next-lower level are gated by the striatum to influence downstream processing. Output gating entails the transition of maintained WM contents from an inert to an action-oriented state—akin to the notion of moving items into the focus of attention.

It is important to note that the involvement of higher-order control mechanisms in WM selection does not make cue-based retrieval obsolete. The memory contents are likely still accessed through the cued contextual features. In support of this notion, prior research has shown that when cues indicate the spatial location of a target object whose color needs to be reported, neural activity related to the spatial location emerges before changes in color-specific activity are evident. Notably, however, both types of information appear earlier in higher-order prefrontal regions than in lower-order sensory areas (Panichello and Buschman, 2021).

### Implications for multi-item working memory access: the role of item and context competition

Although both the interference model and the gating framework are, in principle, applicable to the selection of sets of multiple items, previous research has predominantly focused on single-item retrieval. The WM gating framework predicts that selecting more items should take longer on average because of competitive interactions among higher-order context representations, lower-order WM representations, and possible crosstalk between concurrently active cortico-striatal loops (e.g., Chatham et al., 2014; Desimone and Duncan, 1995; Frank and Badre, 2012; Haber, 2016; Shipp, 2017). Likewise, the interference model implies, though less explicitly, that multi-item retrieval may be less efficient than single-item retrieval to the extent that it involves greater representational overlap and retrieval competition. However, implementations of the model have so far been restricted to single-item access scenarios.

The relatively few studies that have explicitly examined the selection of multiple items from WM generally agree that it is associated with slower responses and reduced accuracy (Gilchrist and Cowan, 2011; Oberauer and Bialkova, 2009, 2011; Tiferet-Dweck et al., 2025). This performance cost has been linked to limits on parallel retrieval of multiple memory elements into the focus of attention, which increases the risk of their confusion or blending—particularly when items are not highly distinct. At the same time, it has been argued that such limits can also be functional rather than structural (e.g., Oberauer and Hein, 2012). For instance, when two items produce different outcomes for a given cognitive operation (e.g., one matches a memory probe while the other does not), holding both in the focus of attention may create crosstalk and complicate decision-making. It has been proposed that, in order to avoid such detrimental consequences, multiple relevant items are often accessed sequentially (Oberauer, 2013).

Beyond these functional limitations, structural limits on multi-item retrieval may also arise from the number of cued *contexts* guiding item selection. Activating multiple contexts simultaneously is likely to amplify the issues arising from imprecision in item-context bindings, producing greater competition and interference at the item-level than in single-item access. If so, retrieval should be more efficient when multiple items can be accessed through a single, shared context rather than through distinct, item-specific contexts (e.g., when several objects share the same spatial location or other retrieval-relevant characteristics). In contrast, competitive interactions or conflict between distinct contexts could be one of the factors that drive multi-item retrieval to proceed sequentially rather than in parallel. Importantly, the initiation of sequential retrieval may itself depend on higher-order control mechanism, such as those posited by the WM gating framework, whose engagement would add another layer of complexity to the selection process. Thus, the cost of selecting multiple items from WM may reflect, at least in part, the need to manage multiple cued contexts.

Crucially, the precise locus of the bottleneck in multi-item access remains uncertain. If limitations arise primarily from the superposition of multiple items in the focus of attention, it should make little difference whether the items are retrieved based on a shared context representation or based on distinct contexts. In contrast, if the main bottleneck is located at the context level, retrieval of multiple items based on a single, shared context should be more efficient. Previous studies have not been able to adjudicate between these possibilities because they typically used paradigms in which each target item was linked to a unique retrieval context (e.g., each memory item was presented in a unique spatial location, which later served as retrieval cue). Consequently, the number of relevant contexts was fully confounded with the number of relevant items.

### The present study

In the present study, we addressed this limitation through a series of experiments that independently manipulated the number of (a) cued contexts (single-context vs. dual-context) and (b) relevant items (single-target vs. dual-target). We used a retrocuing paradigm that was adapted from our previous work on multi-item access (Tiferet-Dweck et al., 2025). Consistent with most prior retrocuing studies, “context” was defined as spatial location. The basic task structure was identical across all experiments: participants encoded sets of three randomly chosen digits that were shown sequentially, each appearing in one of three possible positions within a horizontal grid. The digits were followed by a spatial cue that highlighted either one or two grid locations (single- vs. dual-context), indicating which items participants had to select. Importantly, within each sequence, two digits could appear in the same location, allowing both single- and the dual-context cues to mark either one (single-target trial) or two (dual-target trial) digits as relevant. Thus, when two positions were cued, it was possible that one of them had not been used in the current sequence. To examine the performance cost of selecting an additional item while holding the number of cued spatial contexts constant, we compared (i) single-context/single-target vs. single-context/dual-target trials and (ii) dual-context/single-target vs. dual-context/dual-target trials. Conversely, to test the cost of activating an additional spatial context while holding the number of targets constant, we contrasted (i) single-context/single-target vs. dual-context/single-target trials and (ii) single-context/dual-target vs. dual-context/dual-target trials.

If context-level competition is a major bottleneck, WM access should be slower and less accurate when two spatial locations are cued (dual-context) compared to when only one location is cued (single-context)—both for single-target and dual-target trials. Thus, readiness RTs were expected to be slower for (i) dual-context/single-target trials compared to single-context/single-target trials, and (ii) dual-context/dual-target trials compared to single-context/dual-target trials. Conversely, if the cost is linked to the number of target items, readiness RTs should increase from single-target to dual-target trials. That is, responses should be slower for (i) single-context/dual-target trials compared to single-context/single-target trials and (ii) dual-context/dual-target trials compared to dual-context/single-target trials. Finally, if context- and item-level effects interact in a way that amplifies their combined effect, readiness RTs should be disproportionally slower for dual-context/dual-target relative to all other conditions.

## Experiment 1a

In Experiment 1a, building on our previous work, we aimed to assess how competition at the context and item levels affects the speed of WM selection while also minimizing confounds from probe processing (cf. Tiferet-Dweck et al., 2025). In most retrocuing tasks, performance is measured via responses to a probe display. However, since prior research suggests that WM retrieval and probe comparison are at least partially dissociable processes (Shepherdson et al., 2018), our objective here was to isolate WM access from probe-related processing. To this end, participants were instructed to press a response key as soon as that they had finished item selection following the retrocue. The corresponding reaction time served as our primary measure of WM selection speed. After this initial response, memory for the cued items was probed in a recognition test. The probe was a single digit that either matched one of the relevant digits (target probe) or did not. Non-target probes could be either an uncued digit from the current set (same-trial lure) or a digit that had not appeared in the current trial (other-trial lure). Critically, rejecting same-trial lures required memory for item-context bindings (binding memory), whereas rejecting other-trial lures could rely on item memory alone—the ability to distinguish between items from the current set and those from previous trials (Oberauer, 2005, 2019). Including same-trial lures ensured that participants prioritized only the cued items rather than indiscriminately retaining all three items in each set. Thus, recognition test performance reflected not only selection accuracy but also task compliance.

Because memory set size was limited to three items, overall WM load was low. We therefore expected the number of cued spatial contexts and relevant items to primarily affect the speed of WM selection (readiness RT), with only modest effects on recognition accuracy. Consistent with our previous findings (Tiferet-Dweck et al., 2025), accuracy differences were expected to emerge only for same-trial lures, i.e., when binding memory was required. No systematic accuracy modulations were expected for other-trial lures, i.e., when participants could rely on item memory alone.

Finally, probe reaction times served mainly as a manipulation check. If participants used the retrocue to deprioritize irrelevant items, probe responses should be faster when the relevant was reduced to a single item (single-target) compared to two items (dual-target)—reflecting the well-documented *relevant set-size effect* (Oberauer, 2001, 2018). Importantly, probe RT did not index speed of WM selection itself, but its downstream effect on the efficiency of probe recognition.

### Methods

#### Participants

A total of 65 young adults participated in Experiment 1a. We aimed for an effective sample size of approximately *n* = 40. Based on our previous online studies using similar task paradigms, we expected that roughly one third of datasets would be unusable due to data quality issues. Power estimates were calculated using the R package *mixedpower* (v0.1.0; Kumle et al., 2021), drawing on data from our previous study to derive effect size estimates (Tiferet-Dweck et al., 2025). Participants were recruited from an introductory psychology class at Queens College and received course credit for partaking in the study. All participants were native English speakers and reported normal or corrected-to-normal vision and hearing. Data from 22 participants were excluded because of chance level performance in at least one of the experimental conditions (recognition test accuracy ≤ 50%). An additional three datasets were discarded due to excessively slow responses (RTs > 6s on more than 10 trials). The final sample included 40 datasets, the mean age was 21.4 years (*SD* = 5.34), ranging from 18 to 44 years. Thirty-two participants were female, eight were male (see Supplementary methods for racial/ethnic composition). Participants gave informed consent prior to the experiment and were debriefed upon completion. All experimental procedures were approved by the Queens College Institutional Review Board.

#### Apparatus

All experimental tasks in this study were created using Labvanced^®^ online experiment software (Finger et al., 2017). Participants used their personal devices (laptop or PC) to complete the task. The device needed to be equipped with a display measuring at least 13 inches and a minimum resolution of 800 x 600 pixels, speakers, and a US keyboard.

#### Design

**Figure 1:**
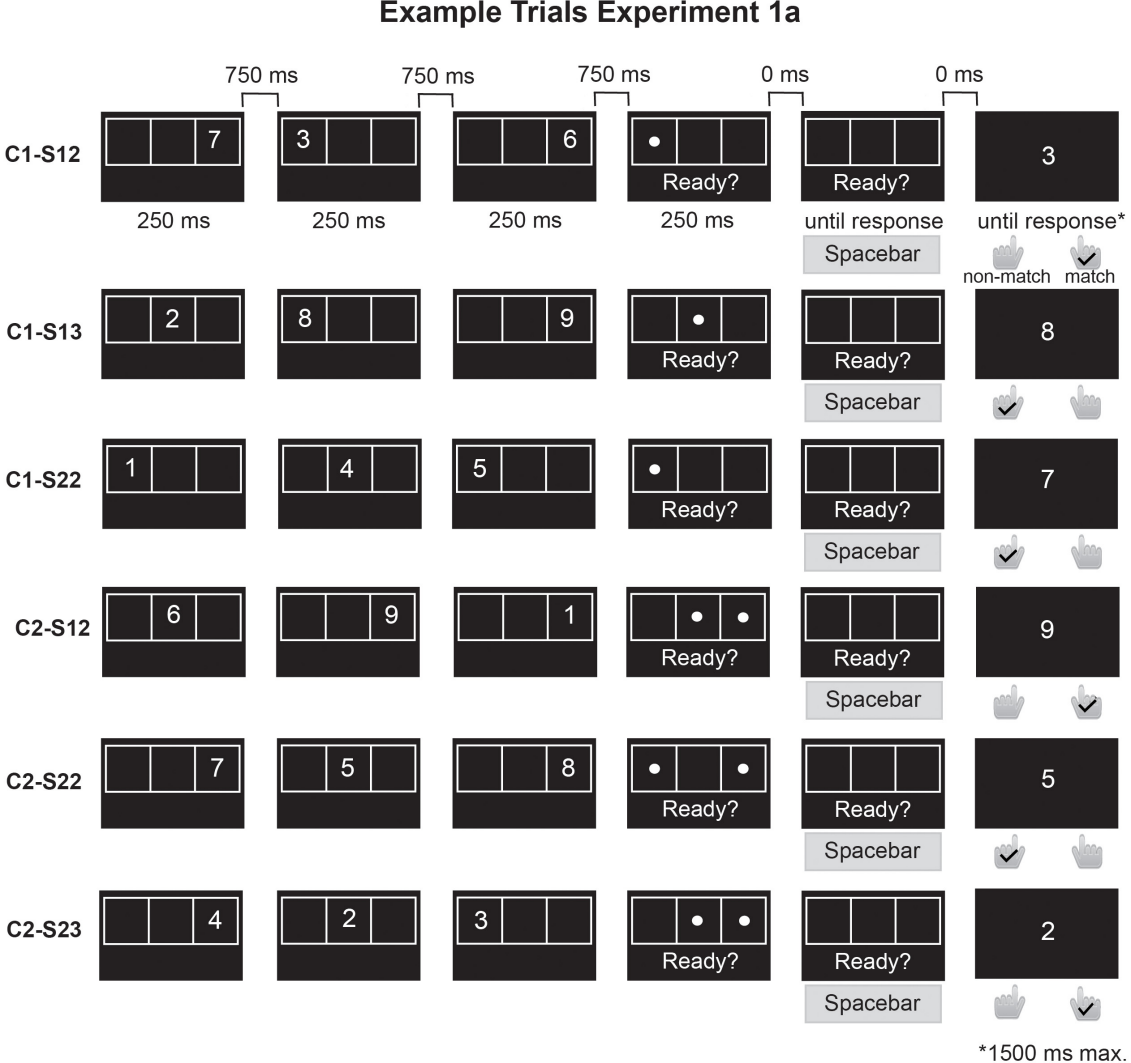
Overview of trial procedures for Experiment 1a. In Experiment 1a, the participants viewed a sequence of three digits that appeared in different locations of a 3 × 1 grid. After the sequence, a spatial retrocue—consisting of one or two dots—indicated which location(s) were relevant. Participants selected the digits that were shown in the cued location(s) and pressed the spacebar when ready. Immediately afterwards, a single probe digit appeared on the screen and the participants decided whether it matched one of the selected (relevant) digits. In the figure, the right key is used for “match” responses and the left key for “non-match” responses (the key assignment was counterbalanced across participants). Probes could be a same-trial lure (digit from an uncued location), a target (digit from a cued location), or an other-trial lure (digit not shown in the current trial). C1, single context; C2, dual context; S1, single-target; S2, dual-target. Only in the single-context/single-target/3loc and dual-context/dual-target/3loc conditions, all three grid locations were used. In all other conditions, one location was used twice, i.e., only two unique locations were used per trial.

We used a 2 x 2 within-subjects design, with the factors *Cue Type* (single- vs. dual context cue) and *Relevant Set Size* (single- vs. dual-target). The corresponding conditions were as follows: single-context/single-target, single-context/dual-target, dual-context/single-target, and dual-context/dual-target. In the single-context/dual-target condition, a single spatial location was associated with two items, that is, two digits appeared in the same grid position. In the dual-context/single-target condition, although two locations were cued, only one contained a digit whereas the other one was unused. Thus, in both single-context/dual-target and dual-context/single-target conditions, the three items in each set appeared in only two distinct spatial locations. In contrast, the single-context/single-target and dual-context/dual-target conditions allowed for the three items to appear in either two or three distinct positions. To account for the number of locations that were used in each trial, the experimental conditions were thus further divided into *single-context/single-target/3loc, single-context/single-target/2loc, single-context/dual-target/2loc, dual-context/single-target/2loc, dual-context/dual-target/2loc*, and *dual-context/dual-target/3loc*, with the digit in the condition names indicating whether items appeared in two or three distinct spatial locations. For instance, in the single-context/single-target-3loc condition, one location was cued (single-context), one digit was relevant (single-target), and each digit was shown in a unique location, i.e., all three grid locations were used (3loc). By contrast, in the single-context/single-target-2loc condition, while the number of spatial contexts and targets was the same, digits appeared only in two distinct locations, i.e., one location was used twice (for an overview, see Figure 1). The task included 48 trials per condition (288 trials total) that were presented in randomized order.

Three dependent variables were assessed: *Readiness RT, Probe Response RT*, and *Probe Response Accuracy*. Readiness RT was used as a proxy of the speed of selection from WM, probe response accuracy measured the correctness of the selection process, and probe response RT indicated how efficiently participants used the context cue to deprioritize the irrelevant items.

#### Stimuli and procedure

Stimuli were presented in light-gray font against a black background. At the beginning of each trial, an empty horizontal 3 × 1 grid was displayed at the center of the screen for a variable 750–1,000 ms interval. Afterward, a sequence of three digits (1–9) was shown, with each digit appearing for 250 ms in one of the grid locations. The digits were separated by intervals of 750 ms. The grid subtended a visual angle of approximately 10.5° × 3.5° and the digits extended over a visual angle of roughly 2.5° × 2.5°, depending on the actual viewing distance. Digits were randomly selected without replacement on each trial, and their spatial positions were also randomly assigned. In the/*2loc* conditions, one grid location was used twice within the same sequence, while in the/*3loc* conditions, each position was used only once. Following the three digits (after another 750 ms interval), one or two dots appeared for 250 ms in randomly chosen grid locations. The dots served as context cues to indicate which of the previously presented digits were relevant for that trial. Simultaneously with the onset of the dot(s), the phrase “Ready?” was displayed beneath the grid, prompting participants to select the cued digits from WM. This readiness prompt remained on the screen until participants pressed the space bar with their thumb, signaling that they had finished retrieval of the relevant items. The time from cue onset to this keypress was recorded as the Readiness RT. Immediately afterward, a recognition probe appeared in the center of the screen. The probe was a single digit and fell into one of three categories. On 50% of trials, the probe was a *target*, meaning it matched one of the relevant digits. On another 25% of trials, it was a *same-trial lure*, a digit from the current trial that had not been cued. On the remaining 25% of trials, the probe was an *other-trial lure*, a digit that had not appeared in the current sequence. When two digits were cued, the target probe was randomly selected among those two, and the uncued digit served as same-trial lure. When only one digit was cued, the same-trial lure was randomly chosen from the two uncued items. Other-trial lures were randomly drawn from the digits not used in the current trial. Participants indicated whether the probe matched one of the relevant digits by pressing the “F” key with their left index finger or the “J” key with their right index finger. The key assignment for match vs. non-match responses was counterbalanced across participants. The deadline for the probe response was set to 1,500 ms. By putting participants under slight time pressure, we tried to prevent them from responding to the preceding readiness prompt before they had completed item selection.

At the beginning of the experimental session, participants filled out a brief demographic questionnaire and watched instructional videos explaining the task. This was followed by a 20-trial practice phase. If they did not achieve a minimum at least 70% accuracy, they were given the option to either exit the study or repeat the practice phase up to two more times. The main task comprised nine blocks that were separated by self-paced breaks. Throughout the task, participants were reminded to keep their fingers in the correct position on the keyboard and to respond as quickly and accurately as possible. The entire session took approximately 50–60 min to complete.

#### Data analysis

All analyses for this study were performed using R version 4.4.0 (R Core Team, 2024). In a first step, the first five trials of the task as well as trials with responses faster than 200 ms or slower than 8 s were excluded. In a second step, trials were excluded if the RT exceeded the individual mean for each condition by more than three *SD*s. For the RT analysis, error trials were also excluded. Accuracy rates were analyzed using generalized mixed-effects models with a logit link function (function *glmer*, R package *lme4* v1.1-35.3; Bates et al., 2015), while RTs were analyzed using linear mixed-effects models (function *lmer*). De*g*rees of freedom were approximated using the Satterthwaite method (R package *lmerTest*, v3.1-3; Kuznetsova et al., 2017). Responses to the readiness prompt (RT only) and the probe (RT and accuracy) were analyzed separately. The RT model for the readiness prompt data included the fixed effect of *selection demand*, with the six levels single-context/single-target/3loc, single-context/single-target/2loc, single-context/dual-target/2loc, dual-context/single-target/2loc, dual-context/dual-target/2loc, and dual-context/dual-target/3loc. The models for probe responses included fixed effects for *selection demand* and *probe type* (target, other-trial lure, same-trial lure). All models were initially set up to include participant-wise random intercepts and random slopes for the fixed effects. In case the data did not support the random slopes for each within-subjects main effect, the random-effects structure was simplified following the model selection criteria suggested by (Bates et al. 2015) and Matuschek et al. (2017). Effect coding was used for all predictors. To determine whether main effects and interactions improved model fit, we compared the full model containing the effect of interest with a reduced model that excluded the effect of interest (using function *anova*). Model fit was evaluated using both the Akaike information criterion (AIC; Akaike, (1974) and Bayes information criterion (BIC; Schwarz, 1978). Standardized model parameters are reported and were calculated using the *parameters* package (Lüdecke et al., 2020). The function *confint* from the *stats* package was used to calculate likelihood profile confidence intervals for all parameter estimates. In contrast, the error bars in all figures represent within-subjects confidence intervals (95%), estimated using parametric bootstrapping (percentile-based intervals) as implemented by the function *bootMer* from the *lme4* package. Significant main effects and interactions were followed up with custom *post-hoc* comparisons using the *emmeans* package (v1.10.1, Lenth, 2024). Šidák correction was used to control the family-wise error rate.

### Results

#### Readiness RT

**Figure 2:**
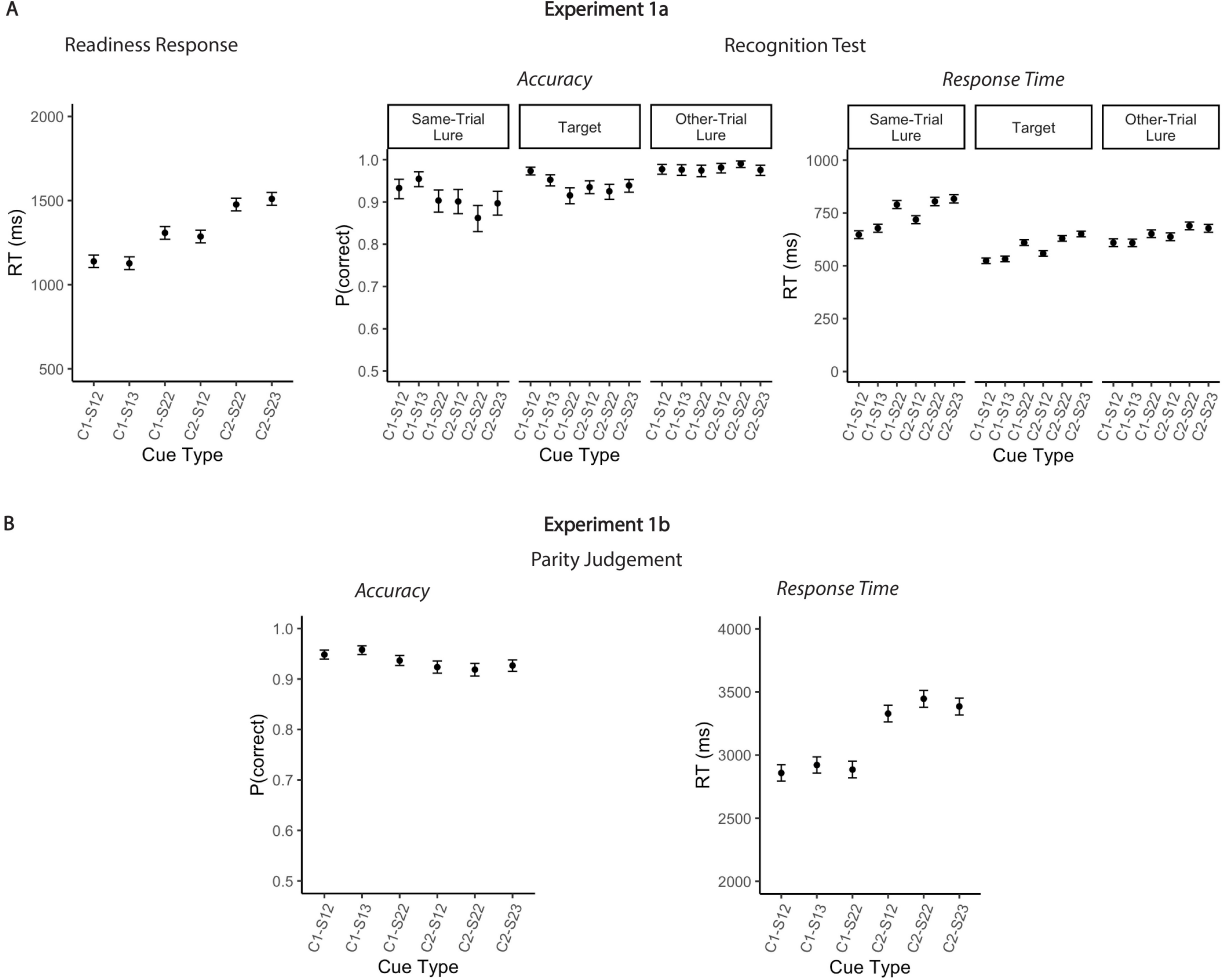
Mean Accuracy rates and reaction times for Experiments 1a and 1b. **(A)** Left: Mean response times to readiness prompt as a function of cue type in Experiment 1a. Right: Mean accuracies and responses times for the recognition test as a function of cue type and probe type in Experiment 1a. **(B)** Mean accuracies **(left)** and responses times **(right)** for the parity judgement (Experiment 1b). Error bars represent 95% within-subject confidence intervals. C1, single context; C2, dual context; S1, single-target; S2, dual-target.

Speed of selection from WM, as indicated by readiness RTs, varied across conditions. Model comparisons confirmed that the full model fit significantly better than the intercept-only model [χ^2^(5) = 350.48, *p* < 0.001, Δ AIC = 440, Δ BIC = 304; see Table 1a]. Readiness RTs were slower when two locations were cued compared to when only one location was cued (Figure 2A). Notably, this RT difference was observed both for single-target trials [single-context/single-target/2loc vs. dual-context/single-target/2loc: *M*_*diff*_ = 148 ms, 95% CIs [74, 222], *t*_(10107.1)_ = 5.45, *p* < 0.001] and dual-target trials [dual-context/dual-target/2loc vs. single-context/dual-target/2loc: *M*_*diff*_ = 169 ms, 95% CIs [95, 244], *t*_(10107.1)_ = 6.18, *p* < 0.001]. Further, RTs increased with the number of relevant items, with slower responses in the single-context/dual-target/2loc compared to the single-context/single-target/2loc condition [*M*_*diff*_ = 169 ms, 95% CIs [95, 243], *t*_(10107.1)_ = 6.21, *p* < 0.001] as well as in the dual-context/dual-target/2loc compared to the dual-context/single-target/2loc condition [*M*_*diff*_ = 190 ms, 95% CIs [116, 265], *t*_(10107.1)_ = 6.95, *p* < 0.001]. There was no evidence for an interaction of cue type and relevant set size—the RT difference between single-context and dual-context conditions was comparable across single- vs. dual-target trials [*M*_*diff*_ = 22 ms, 95% CI [−83, 127], *t*_(10107.1)_ = 0.56, *p* = 1.00]. Moreover, the effect of the number of cued spatial contexts on RTs was comparable to the effect of relevant items, i.e., the RT differences between (i) single-context and dual-context trials (averaged across number of targets) and (ii) single-target and dual-target trials (averaged across number of cued contexts) did not differ significantly [*M*_*diff*_ = 21 ms, 95% CI [−83, 127], *t*_(10107.1)_ = 0.76, *p* = 0.99]. Finally, RTs did not differ significantly depending on whether the items in a given set occupied two vs. three distinct grid locations, provided all other factors were held constant [single-context/single-target/3loc vs. single-context/single-target/2loc: *M*_*diff*_ = 12 ms, 95% CIs [−61, 85], *t*_(10107.1)_ = 0.45, *p* = 1.00; dual-context/dual-target/2loc vs. dual-context/dual-target/3loc: *M*_*diff*_ = 33 ms, 95% CIs [−42, 108], *t*_(10107.1)_ = 1.21, *p* = 0.87].

**Table 1a:** Model fit and *post-hoc* comparisons for readiness prompt (Experiment 1a).

Full model: AIC = 164,336, BIC = 164,394, ***${\text{R}}_{adj}^{2}$*** = 0.303					
Intercept-only model: AIC = 164,676, BIC = 164,698, *$R_{adj}^{2}$* = 0.278					
Model comparison: *χ^2^(5)* = 350.48, *p* < 0.001					
**Contrast**	**Estimated mean difference**	***df***	***t*** **-value**	***p*** **-value**	**95% CI**
C1-S12 vs. C1-S13	12	10107.1	0.45	1.00	[−61, 85]
C1-S12 vs. C1-S22	−169	10107.1	−6.21	< 0.001	[−243, −95]
C1-S12 vs. C2-S12	−148	10107.1	−5.45	< 0.001	[−222, −74]
C1-S22 vs. C2-S22	−169	10107.1	−6.18	< 0.001	[−244, −95]
C2-S12 vs. C2-S22	−190	10107.1	−6.95	< 0.001	[−265, −116]
C2-S22 vs. C2-S23	−33	10107.1	−1.21	0.87	[−108, 42]
(C1-S12 to C1-S22) vs. (C2-S12 to C2-S22)	22	10107.1	0.56	1.00	[−83, 127]
(Cx-S12 to Cx-S22) vs. (C1-Sx to C2-Sx)^a^	21	10107.1	0.76	0.99	[−54, 96]

CI, confidence interval; C1, single context; C2, dual context; S1, single-target; S2, dual-target.

^a^This contrast tested whether the effect of relevant set size, averaged across C1 and C2 cues, is different from the effect of number of cued contexts, averaged across both relevant set sizes.

#### Recognition test

##### Accuracy

Response accuracy in the recognition test was influenced by both selection demands [χ^2^(5) = 51.97, *p* < 0.001, Δ AIC = 42, Δ BIC = 6] and probe type [χ^2^(2) = 166.32, *p* < 0.001, Δ AIC = 163, Δ BIC = 148; see Table 1b]. Statistical evidence for an interaction between these two factors was weak, as indicated by an increase in BIC when the interaction term was included [χ^2^(10) = 29.92, *p* < 0.001, Δ AIC = 10, Δ BIC = −63]. As can be seen from Figure 2A, performance was worse for same-trial lures compared to both target probes [OR = 0.63, 95% CI [0.48, 0.83], *z* = −5.29, *p* < 0.001] and other-trial lures [OR = 0.21, 95% CI [0.14, 0.33], *z* = −11.00, *p* < 0.001]. Accuracy was also lower for targets than for other-trial lures [OR = 0.34, 95% CI [0.22, 0.51], *z* = −8.00, *p* < 0.001]. When one location was cued, participants committed slightly fewer errors on single-target than dual-target trials [single-context/single-target/2loc vs. single-context/dual-target/2loc: OR = 1.78, 95% CI [1.06, 2.98], *z* = 3.48, *p* = 0.013]. This effect was driven exclusively by target probes [OR = 3.37, 95% CI [1.72, 6.58], *z* = 5.65, *p* < 0.001], with no significant differences observed for either same-trial lures [OR = 1.47, 95% CI [0.72, 3.02], *z* = 1.68, *p* = 0.93] or other-trial lures [OR = 1.14, 95% CI [0.34, 3.79], *z* = 0.34, *p* = 1.00]. Performance did not vary significantly based on relevant set size when two locations were cued [dual-context/single-target/2loc vs. dual-context/dual-target/2loc: OR = 0.97, 95% CI [0.53, 1.75], *z* = −0.17, *p* = 1.00]. No other effects reached significance.

**Table 1b:** Model fit and *post-hoc* comparisons for probe response (Experiment 1a).

**Accuracy**				
Full model: AIC = 5,057, BIC = 5,196, *$R_{adj}^{2}$* = 0.253				
Reduced model (both main effects): AIC = 5,067, BIC = 5,133, *$R_{adj}^{2}$* = 0.243				
Reduced model (main effect probe type only): AIC = 5,109, BIC = 5,139, *$R_{adj}^{2}$* = 0.226				
Reduced model (main effect selection demand only): AIC = 5,230, BIC = 5,281, *$R_{adj}^{2}$* = 0.175				
Model comparison (full model vs. no interaction): *χ^2^(10)* = 29.92, *p* < 0.001				
Model comparison (both main effects vs. probe type only): *χ^2^(5)* = 51.97, *p* < 0.001				
Model comparison (both main effects vs. selection demand only): *χ^2^(2)* = 166.32, *p* < 0.001				
**Contrast**	**Odds ratio**	***z*** **-value**	***p*** **-value**	**95% CI**
Same-trial lure vs. other-trial lure	0.21	−11.00	< 0.001	[0.14, 0.33]
Target vs. other-trial lure	0.34	−8.00	< 0.001	[0.22, 0.51]
Same-trial lure vs. target	0.63	−5.29	< 0.001	[0.48, 0.83]
C1-S12 vs. C1-S13	1.08	0.45	1.00	[0.63, 1.87]
C1-S12 vs. C1-S22	1.78	3.48	0.013	[1.06, 2.98]
C1-S12 vs. C2-S12	1.47	2.20	0.53	[0.85, 2.52]
C1-S22 vs. C2-S12	0.82	−1.20	1.00	[0.50, 1.36]
C1-S22 vs. C2-S22	0.80	−1.23	1.00	[0.45, 1.41]
C2-S12 vs. C2-S22	0.97	−0.17	1.00	[0.53, 1.75]
C2-S22 vs. C2-S23	0.88	−0.72	1.00	[0.49, 1.55]
Same-trial lure: C1-S12 vs. C1-S13	0.65	−1.57	0.96	[0.28, 1.62]
Other-trial lure: C1-S12 vs. C1-S13	1.06	0.15	1.00	[0.31, 3.59]
Target: C1-S12 vs. C1-S13	1.83	2.61	0.22	[0.89, 3.77]
Same-Trial Lure: C1-S12 vs. C1-S22	1.47	1.68	0.93	[0.72, 3.02]
Other-trial lure: C1-S12 vs. C1-S22	1.14	0.34	1.00	[0.34, 3.79]
Target: C1-S12 vs. C1-S22	3.37	5.65	< 0.001	[1.72, 6.58]
Same-trial lure: C1-S12 vs. C2-S12	1.51	1.79	0.88	[0.74, 3.08]
Other-trial lure: C1-S12 vs. C2-S12	0.83	−0.44	1.00	[0.23, 3.03]
Target: C1-S12 vs. C2-S12	2.53	4.18	< 0.001	[1.27, 5.04]
Same-trial lure: C1-S22 vs. C2-S22	1.50	2.00	0.73	[0.90, 2.81]
Other-trial lure: C1-S22 vs. C2-S22	0.38	−1.97	0.76	[0.08, 1.75]
Target: C1-S22 vs. C2-S22	0.88	−0.78	1.00	[0.53, 1.46]
Same-trial lure: C2-S12 vs. C2-S22	1.46	1.90	0.81	[0.78, 2.74]
Other-trial lure: C2-S12 vs. C2-S22	0.53	−1.26	1.00	[0.11, 2.58]
Target: C2-S12 vs. C2-S22	1.18	0.94	1.00	[0.69, 2.01]
Same-trial lure: C2-S22 vs. C2-S23	0.72	−1.65	0.95	[0.39, 1.34]
Other-trial lure: C2-S22 vs. C2-S23	2.56	1.94	0.78	[0.56, 11.68]
Target: C2-S22 vs. C2-S23	0.81	−1.23	1.00	[0.47, 1.39]
**RT**				
Full model: AIC = 13,6248, BIC = 136,393, *$R_{adj}^{2}$* = 0.368				
Reduced model (both main effects): AIC = 136,278, BIC = 136,350, *$R_{adj}^{2}$* = 0.366				
Reduced model (main effect probe type only): AIC = 136,832, BIC = 136,868, *$R_{adj}^{2}$* = 0.330				
Model comparison (both main effects vs. probe type only): *χ^2^(5)* = 563.71, *p* < 0.001				
Model comparison (both main effects vs. selection demand only): *χ^2^(2)* = 987.65, *p* < 0.001				
**Contrast**	**Estimated mean difference**	***df***	***t*** **-value**	***p*** **-value**	**95% CI**
Same-trial lure vs. other-trial lure	97	10119.3	17.48	< 0.001	[80, 114]
Target vs. other-trial lure	−62	10119.2	−13.05	< 0.001	[−76, −47]
Same-trial lure vs. target	159	10119.3	32.28	< 0.001	[143, 174]
C1-S12 vs. C1-S13	−13	10119.1	−1.81	0.87	[−35, 9]
C1-S12 vs. C1-S22	−90	10119.1	−12.56	< 0.001	[−112, −68]
C1-S12 vs. C2-S12	−45	10119.1	−6.22	< 0.001	[−67, −22]
C1-S22 vs. C2-S12	45	10119.1	6.29	< 0.001	[23, 68]
C1-S22 vs. C2-S22	−24	10119.1	−3.39	0.020	[-47,−2]
C2-S12 vs. C2-S22	−70	10119.1	−9.65	< 0.001	[−92, −47]
C2-S22 vs. C2-S23	−7	10119.1	−1.01	1.00	[−30, 15]
Same-Trial Lure: C1-S12 vs. C1-S13	−31	10119.1	−2.25	0.50	[−72, 12]
Other-trial lure: C1-S12 vs. C1-S13	1	10119.1	0.04	1.00	[−41, 42]
Target: C1-S12 vs. C1-S13	−8	10119.1	−0.87	1.00	[−38, 21]
Same-trial lure: C1-S12 vs. C1-S22	−142	10119.2	−10.24	< 0.001	[−185, −99]
Other-trial lure: C1-S12 vs. C1-S22	−42	10119.1	−3.19	0.032	[−83, −1]
Target: C1-S12 vs. C1-S22	−85	10119.2	−8.82	< 0.001	[−115, −55]
Same-trial lure: C1-S12 vs. C2-S12	−71	10119.2	−5.13	< 0.001	[−92, −47]
Other-trial lure: C1-S12 vs. C2-S12	−28	10119.1	−2.12	0.62	[−70, 13]
Target: C1-S12 vs. C2-S12	−34	10119.2	−3.55	0.011	[−64, −4]
Same-trial lure: C1-S22 vs. C2-S22	−15	10119.2	−1.07	1.00	[−60, 29]
Other-trial lure: C1-S22 vs. C2-S22	−38	10119.1	−2.88	0.11	[−79, 3]
Target: C1-S22 vs. C2-S22	−20	10119.1	−2.09	0.65	[−50, 10]
Same-trial lure: C2-S12 vs. C2-S22	−86	10119.1	−6.05	< 0.001	[−131, −42]
Other-trial lure: C2-S12 vs. C2-S22	−52	10119.1	−3.93	0.002	[−93, −11]
Target: C2-S12 vs. C2-S22	−71	10119.1	−7.34	< 0.001	[−102, −41]
Same-trial lure: C2-S22 vs. C2-S23	−12	10119.2	−0.86	1.00	[−57, 32]
Other-trial lure: C2-S22 vs. C2-S23	12	10119.1	0.88	1.00	[−29, 52]
Target: C2-S22 vs. C2-S23	−21	10119.1	−2.17	0.57	[−51, 9]
CI, confidence interval; C1, single context; C2, dual context; S1, single-target; S2, dual-target.

##### RT

Response speed for probe recognition varied significantly as a function of both selection demands [χ^2^(5) = 563.71, *p* < 0.001, Δ AIC = 554, Δ BIC = 518] and probe type [χ^2^(2) = 987.65, *p* < 0.001, Δ AIC = 984, Δ BIC = 969; see Table 1b]. There was only limited evidence for an interaction between these factors [χ^2^(10) = 49.50, *p* < 0.001, Δ AIC = 30, Δ BIC = −43]. Response times were increased for same-trial lures compared to targets [*M*_*diff*_ = 159 ms, 95% CIs [143, 174], *t*_(10119.3)_ = 32.28, *p* < 0.001] and other-trial lures [*M*_*diff*_ = 97 ms, 95% CIs [80, 114], *t*_(10119.3)_ = 17.48, *p* < 0.001; see Figure 2A]. Additionally, participants responded more slowly to other-trial lures than to targets [*M*_*diff*_ = 62 ms, 95% CIs [47, 76], *t*_(10119.2)_ = 13.05, *p* < 0.001]. Response speed was faster for single-target compared to dual-target trials. This effect held regardless of the number of cued locations—whether one location was cued [single-context/single-target/2loc vs. single-context/dual-target/2loc: *M*_*diff*_ = 90 ms, 95% CIs [68, 112], *t*_(10119.1)_ = 12.56, *p* < 0.001] or two [dual-context/single-target/2loc vs. dual-context/dual-target/2loc: *M*_*diff*_ = 70 ms, 95% CIs [47, 92], *t*_(10119.1)_ = 9.65, *p* < 0.001]. The largest RT differences between single- and dual-target trials were found for same-trial lures (*M*_*diff*_ = 142 and 86 ms, for single-context and dual-context conditions, respectively), followed by targets (*M*_*diff*_ = 85 and 71 ms), and other-trial lures (*M*_*diff*_ = 42 and 52 ms; Table 1b). Response speed was also affected by the number of cued spatial contexts. Responses were slower when two locations were cued rather than just one, particularly in the single-target conditions [*M*_*diff*_ = 45 ms, 95% CI [22, 67], *t*_(10119.1)_ = 6.22, *p* < 0.001], where the effect was significant for same-trial lures [*M*_*diff*_ = 71 ms, 95% CIs [29, 114], *t*_(10119.2)_ = 5.13, *p* < 0.001] and targets [*M*_*diff*_ = 34 ms, 95% CIs [5, 64], *t*_(10119.2)_ = 3.55, *p* = 0.009]. However, for dual-target trials, the effect was smaller [dual-context/dual-target/2loc vs. single-context/dual-target/2loc; *M*_*diff*_ = 24 ms, 95% CIs [2, 47], *t*_(10119.1)_ = 3.39, *p* = 0.020], and did not reach significance when tested individually across the different probe types (Table 1b).

### Discussion

Experiment 1a provided evidence that the speed of selecting information from WM is influenced both by the number of items that need to be selected and by the number of spatial locations (i.e., contexts) guiding this selection. We found that readiness RTs increased when two items needed to be selected compared to when only one item was relevant. This slowing occurred regardless of whether one or two locations were cued. Crucially, RTs were also increased when the relevant items were each bound to unique spatial contexts as opposed to a single shared context. This context-based slowing was observed irrespective of the number of selected items, suggesting additive effects of (i) relevant set size and (ii) number of cued locations to delay selection from WM.

Importantly, the RT data for the recognition test indicated that participants did not simply ignore the preceding readiness prompt, supporting the validity of using readiness RT as a measure for the speed of WM selection. Probe responses were faster when participants needed to compare the probe to only one target (single target) rather than two (dual target), indicating that they indeed narrowed the relevant set size to a single item, allowing quicker recognition decisions (relevant set-size effect; Oberauer, 2001, 2018). In contrast, we found little evidence for a similar benefit of smaller relevant set size on response accuracy. Error rates were largely unaffected by the number of relevant items. The only notable exception was a reduction in errors when a single-location cue prompted retrieval of one target rather two targets (single-context/single-target/2loc vs. single-context/dual-target/2loc). Since this improvement occurred only when the probe was a target, it may indicate that participants occasionally selected only one of the two relevant items on single-context/dual-target/2loc trials. Thus, if the non-selected item was probed, it would have been falsely rejected. In contrast, no single-target benefit on accuracy rates was found when two spatial contexts were cued (dual-context/single-target/2loc vs. dual-context/dual-target/2loc). The absence of a robust accuracy improvement for single-target trials resembles the lack of retrocuing benefits that other studies have found when using sequentially presented lists of WM items (Oberauer, 2018). It has been argued that when items are sequentially encoded, retrocues are less effective at shielding memory representations from visual interference, limiting their potential to reduce retrieval errors. Instead, they primarily enable faster access to the target item through advance retrieval—that is, retrieving the target before probe onset—thereby enhancing response speed (Shepherdson et al., 2018).

Interestingly, the number of cued locations also influenced RTs in the recognition test. Specifically, probe responses were slower following dual-context cues compared to single-context cues, especially, when only one item was relevant. This pattern suggests that cueing two locations amplifies intrusion effects from irrelevant item(s) during the subsequent recognition test. One possible explanation is that the additional time required to activate a second spatial context allowed relatively more evidence to accumulate for all items in the memory set, including non-targets. As a result, greater lingering activation of irrelevant items may have slowed the recognition decision.

#### Limitations of Experiment 1a and rationale for Experiments 1b to 2c

Two key limitations tempered the strength of our conclusions from Experiment 1a: First, readiness RTs were based on participants' own judgment of when they had completed the selection of the relevant items from WM. Due to the subjective nature of the underlying processes, this response may have been influenced by factors beyond WM access itself, such as metacognitive monitoring or strategic considerations. This possibility was addressed in Experiment 1b, which included neither readiness nor probe responses but required participants to use the relevant digits as operands in an addition problem.

A second potential limitation of Experiment 1a arises from the probabilistic structure of the task. Specifically, the task paradigm included two contingencies that could have led participants to form expectations about the number and spatial distribution of relevant items. One contingency concerns the likelihood of targets being bound to distinct vs. shared spatial contexts. In the single-context/single-target/3loc, single-context/single-target/2loc, dual-context/single-target/2loc, and dual-context/dual-target/3loc conditions, each target item appeared in a unique spatial location (two-third of trials), whereas in the single-context/dual-target/2loc and dual-context/dual-target/2loc conditions, both targets appeared in the same location (one third of trials). As a result, participants may have expected that each relevant item would occupy its own spatial location. Violations of this expectation—i.e., when two targets had to be retrieved from a single location—could have contributed to longer RTs and/or increased error rates in the single-context/dual-target/2loc and dual-context/dual-target/2loc conditions. Notably, these conditions were central to our conclusion that competition at the item level constrains WM access. Thus, any bias specifically affecting these trials would challenge the validity of this interpretation. Another possible confound is that participants may have come to expect the number of target items to match the number of cued locations. A mismatch—such as two cued locations with only one relevant item (dual-context/single-target/2loc), or one cued location with two relevant items (single-context/dual-target/2loc)—could have introduced greater uncertainty during WM selection and decision-making, leading to increased RTs in those conditions. While this expectation of a one-to-one mapping between cued spatial contexts and target items might reflect an a-priori cognitive bias (and as such would be difficult to control), it could also have emerged from the probabilistic structure of the task. When one location was cued (single-context), single-target trials (single-context/single-target/3loc, single-context/single-target/2loc) were more frequent than dual-target trials (single-context/dual-target/2loc). Conversely, when two locations were cued (dual context), dual-target trials (dual-context/dual-target/2loc, dual-context/dual-target/3loc) were more frequent than those with only one target (dual-context/single-target/2loc). Experiments 2a to 2c were therefore specifically designed to eliminate these inherent task contingencies.

## Experiment 1b

To obtain a more objective measure of WM selection—and to validate the two-stage response procedure used in Experiment 1a—we conducted a follow-up study in which the readiness response was omitted. Participants again viewed sequences of three digits followed by a retrocue indicating the spatial location(s) of the relevant item(s). However, no readiness prompt was presented. Instead, participants were asked to mentally add the cued items and to indicate via button presses whether the resulting sum was odd or even. On single-target trials, participants retrieved one digit from memory and added it to a second digit that was presented beneath the retrocue. On dual-target trials, both operands were retrieved from memory, and the additional digit beneath the cue had to be ignored. Thus, task demands differed between single- and dual-target trials—requiring either encoding of the second operand from perceptual input or its retrieval from WM. For this reason, Experiment 1b was not suited to isolate the effect of relevant set size. Rather, its primary goal was to examine the effect of the number of cued spatial contexts on task performance *within* the single- and dual-target conditions. Specifically, we sought to replicate the finding from Experiment 1a that selection from WM is slower when two contexts are cued compared to one, while holding the number of relevant items constant.

### Methods

#### Participants

Fifty-seven young adults were recruited from Prolific© and received $12 for their participation. Based on power analyses informed by the effect sizes from Experiment 1a, we aimed for an effective sample size of approximately *n* = 40. Seventeen participants were excluded from data analyses due to performing at chance level in one or more conditions (*n* = 16). The effective sample thus included 40 datasets (mean age 28.1 years, *SD* = 4.82, range 19–35 years, 20 females, 20 males; see Supplementary methods for racial and ethnic composition).

#### Design, stimuli, and procedure

**Figure 3:**
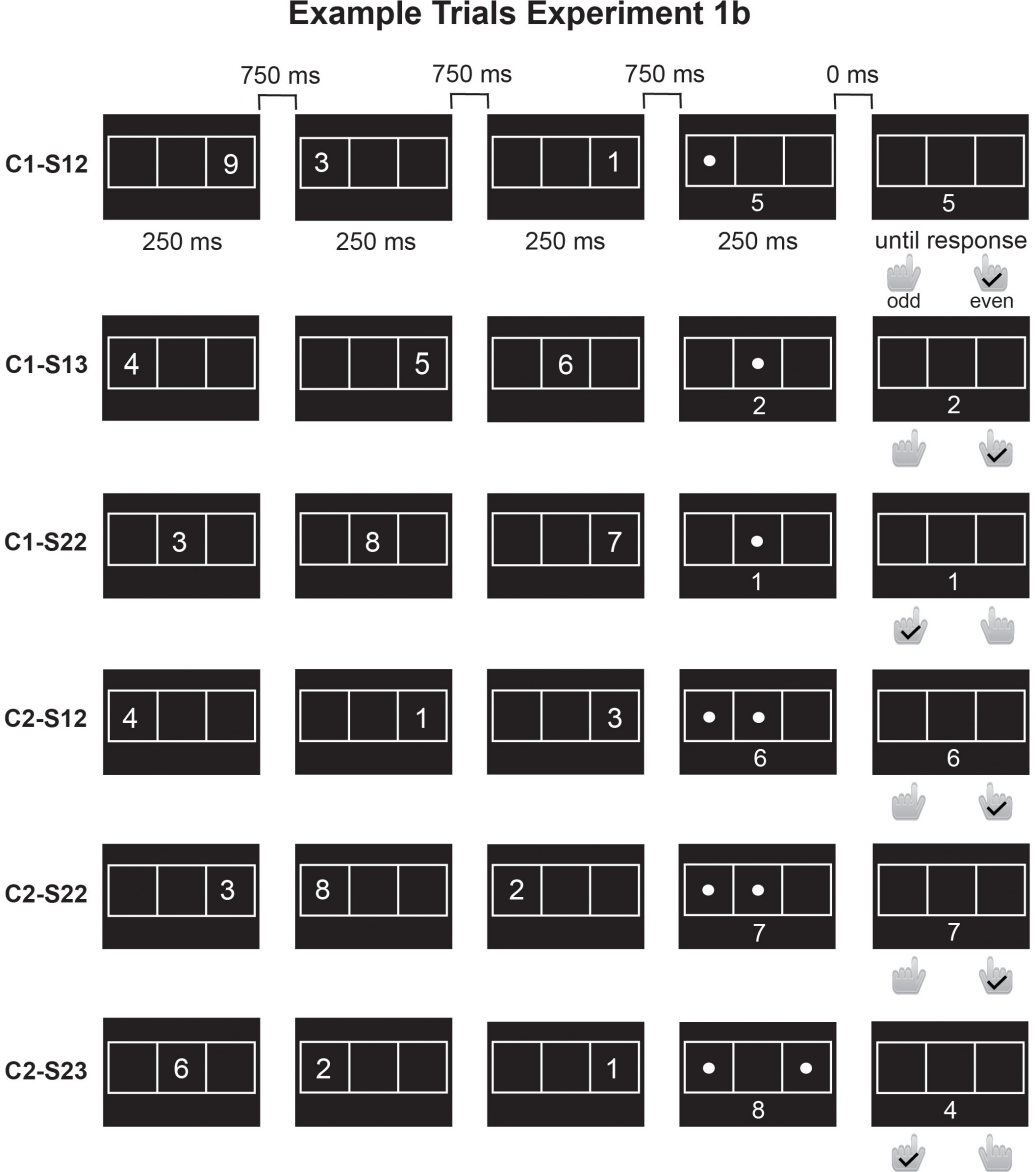
Overview of trial procedures for Experiment 1b. As in Experiment 1a, the participants viewed a sequence of three digits that appeared in different locations of a 3 × 1 grid, followed by a spatial retrocue indicating the relevant locations. Unlike in Experiment 1a, there was no readiness prompt. Instead, a single digit was presented below the retrocue. When one digit was cued (single-context/single-target/2loc, single-context/single-target/3loc, dual-context/single-target/2loc), participants added the cued digit to the one shown below the retrocue. When two digits were cued (single-context/dual-target/2loc, dual-context/dual-target/2loc, dual-context/dual-target/3loc), participants ignored the digit on the screen and added the two cued digits. In both cases, participants pressed the left or right response keys to indicate whether the sum was odd or even. In the example trials, the right key is used for “even” responses, the left key for “odd” responses. C1, single context; C2, dual context; S1, single-target; S2, dual-target.

We used a modified version of the task paradigm from Experiment 1a. All presentation parameters for the digit sequence and retrocue were identical to those described before. However, instead of the readiness prompt, a randomly selected digit (1–9) appeared directly below the retrocue (SOA = 0) and remained on the screen for the remainder of the trial. Participants were instructed to perform a mental addition task using the cued digits as operands. When only one digit was relevant (single-context/single-target/2loc, single-context/single-target/3loc, dual-context/single-target/2loc), participants retrieved the cued digit from memory and added it to the digit displayed beneath the retrocue. When two items were relevant (single-context/dual-target/2loc, dual-context/dual-target/2loc, dual-context/dual-target/3loc), both cued digits served as operands and were added together, while the on-screen digit had to be ignored (see Figure 3). Participants then indicated whether the sum was odd or even by pressing the “F” or “J” keys on their keyboard, respectively. The assignment of the keys to response options was counterbalanced across participants. All other experimental procedures were to those in Experiment 1a.

### Results and discussion

Speed of WM access varied systematically with selection demands. Model comparisons confirmed that the full RT model fit significantly better than the intercept-only model [χ^2^(5) = 335.80, *p* < 0.001, Δ AIC = 326, Δ BIC = 290; see Table 2]. Crucially, pairwise comparisons revealed that RTs were slower when two spatial contexts were cued rather than only one—both for single-target trials [*M*_*diff*_ = 471 ms, 95% CIs [344, 598], *t*_(9666.2)_ = 9.95, *p* < 0.001] and dual-target trials [*M*_*diff*_ = 562 ms, 95% CIs [434, 689], *t*_(10119.1)_ = 11.80, *p* < 0.001; Figure 2B]. These results replicate the findings from Experiment 1a and further support the conclusion that retrieving WM contents based on multiple spatial contexts incurs an RT cost, independent of the number of selected items. We found limited evidence for accuracy differences across conditions [χ^2^(5) = 46.07, *p* < 0.001, Δ AIC = 36, Δ BIC = 0; see Table 2]. *Post-hoc* comparisons showed a modest increase in error rates when two spatial contexts were cued rather than one, but only for single-target trials [OR = 1.51, 95% CI [1.09, 2.10], *z* = 3.38, *p* = 0.005; Figure 2B]. No significant context-related accuracy difference was observed for dual-target trials [OR = 1.31, 95% CI [0.96, 1.79], *z* = 2.34, *p* = 0.13].

**Table 2:** Model fit and *post-hoc* comparisons (Experiment 1b).

**Accuracy**				
Full model: AIC = 5,874, BIC = 5,925, *$R_{adj}^{2}$* = 0.245				
Intercept-only model: AIC = 5,910, BIC = 5,925, *$R_{adj}^{2}$* = 0.233				
Model comparison: *χ^2^(5)* = 46.07, *p* < 0.001				
**Contrast**	**Odds ratio**	***z*** **-value**	***p*** **-value**	**95% CI**
C1-S12 vs. C1-S13	0.81	−1.58	0.57	[0.56, 1.16]
C1-S12 vs. C1-S22	1.24	1.68	0.49	[0.88, 1.73]
C1-S12 vs. C2-S12	1.51	3.38	0.005	[1.09, 2.10]
C1-S22 vs. C2-S12	1.22	0.76	0.99	[0.89, 1.68]
C1-S22 vs. C2-S22	1.31	2.34	0.13	[0.96, 1.79]
C2-S12 vs. C2-S22	1.07	0.62	1.00	[0.79, 1.45]
C2-S22 vs. C2-S23	0.89	−0.99	0.93	[0.66, 1.21]
**RT**					
Full model: AIC = 167,482, BIC = 167,539, *$R_{adj}^{2}$* = 0.460					
Intercept-only model: AIC = 167,808, BIC = 167,829, *$R_{adj}^{2}$* = 0.439					
Model comparison: *χ^2^(5)* = 335.80, *p* < 0.001					
**Contrast**	**Estimated mean difference**	***df***	***t*** **-value**	***p*** **-value**	**95% CI**
C1-S12 vs. C1-S13	−63	9666.1	−1.36	0.74	[−187, 61]
C1-S12 vs. C1-S22	−27	9666.1	−0.58	1.00	[−153, 99]
C1-S12 vs. C2-S12	−471	9666.2	−9.95	< 0.001	[−598, −344]
C1-S22 vs. C2-S12	−444	9666.1	−9.33	< 0.001	[−571, −316]
C1-S22 vs. C2-S22	−562	9666.1	−11.80	< 0.001	[−689, −434]
C2-S12 vs. C2-S22	−118	9666.2	−2.46	0.093	[−247, 11]
C2-S22 vs. C2-S23	62	9666.2	1.30	0.78	[−66, 191]

CI, confidence interval; C1, single context; C2, dual context; S1, single-target; S2, dual-target.

Neither RTs nor accuracy rates differed reliably as a function of relevant set size (Table 2). The absence of RT differences between single- and dual-target trials likely reflects condition-specific task demands: on single-target trials, participants encoded a second operand that was presented on-screen, whereas on dual-target trials, both operands were retrieved from WM. The additional encoding step in the single-target condition may have offset the typical RT advantage associated with smaller relevant set sizes (Ricker et al., 2018). Moreover, single-target trials required participants to coordinate the encoding of a new digit with the retrieval of a maintained one, potentially placing greater demands on executive control. This added level of complexity may also have contributed to the lack of accuracy differences between single- and dual-target trials. Notably, however, we also did not observe clear effects of relevant set size on accuracy in Experiment 1a, suggesting that accuracy measures were generally insensitive to the present task manipulations, possibly owing to the modest WM load (three items) and the sequential nature of the task (see also General Discussion).

Taken together, the current findings are largely consistent with the results from Experiment 1a, aside from predictable performance differences due to variations in task demands. Most importantly, the results indicate that number of cued spatial contexts slows selection from WM, even when the number of relevant items is held constant. Although the task design did permit a simultaneous test of item-number effects, the findings support the notion that readiness RT in Experiment 1a provides a sensitive index of WM retrieval speed.

## Experiments 2a and 2b

Experiments 2a and 2b tested whether performance in the previous two experiments may have been influenced by the probabilistic structure of the task. Specifically, we examined the potential effects of two inherent contingencies. First, only in the single-context/dual-target/2loc and dual-context/dual-target/2loc conditions, both targets were bound to a shared spatial context, whereas in the remaining four conditions (single-context/single-target/2loc, single-context/single-target/3loc, dual-context/single-target/2loc, dual-context/dual-target/3loc), targets were bound to distinct contexts. As a result, a shared context was less expected, possibly leading to slower responses in the single-context/dual-target/2loc and dual-context/dual-target/2loc conditions. This imbalance was removed by dividing the original set of six conditions into two partially overlapping subsets of four conditions each. Specifically, Experiment 2a included only single-context/single-target/3loc, single-context/dual-target/2loc, dual-context/dual-target/2loc, and dual-context/dual-target/3loc trials, whereas Experiment 2b included only single-context/single-target/2loc, single-context/dual-target/2loc, dual-context/single-target/2loc, and dual-context/dual-target/2loc trials. Consequently, the proportions of trials in which the targets were bound to distinct vs. shared contexts were fully balanced in both experiments. In Experiment 2a, targets were shown in distinct locations in half of the trials (single-context/single-target/3loc, dual-context/dual-target/3loc) and in a shared location in the other half (single-context/dual-target/2loc, dual-context/dual-target/2loc). Similarly, in Experiment 2b, half of the trials contained targets with unique contexts (single-context/single-target/2loc, dual-context/single-target/2loc), whereas in the other half, targets shared a context (single-context/dual-target/2loc, dual-context/dual-target/2loc).

Second, participants may have developed an expectation that the number of cued locations would match the number of relevant items, potentially affecting their performance when this expectation was violated—as in the single-context/dual-target/2loc and dual-context/single-target/2loc conditions. The impact of this contingency was tested in Experiment 2b which fully decoupled the number of cued locations from the number of relevant items. Regardless of whether one (single-context) or two (dual context) locations were cued, exactly half of the trials contained one target item and half contained two, thereby eliminating the predictive relationship.

### Methods

#### Participants

A total of 113 young adults participated in Experiment 2a (*n* = 61) and 2b (*n* = 52). Participants were recruited from an introductory psychology course at Queens College (Experiment 2a) and via Prolific© (Experiment 2b). In Experiment 2a, 15 participants were excluded from data analyses due to low accuracy (*n* = 13) or slow responses (*n* = 2). The final sample for Experiment 2a thus included 46 datasets (mean age 19.9 years, *SD* = 2.40, range 18–30 years; 28 females, 18 males; see Supplementary methods for racial and ethnic composition). In Experiment 2b, data from one participant were excluded due to chance level performance. The final sample included 51 datasets, the mean age was 29.0 years (*SD* = 5.26), ranging from 19 to 48 years. Thirteen participants were female, 37 were male, one self-identified as non-binary/other gender (see Supplementary methods for racial and ethnic composition).

#### Design, stimuli, and procedure

All stimuli and procedures were identical to those in Experiment 1a, except for the following changes. Experiment 2a comprised only the conditions single-context/single-target/3loc, single-context/dual-target/2loc, dual-context/dual-target/2loc, and dual-context/dual-target/3loc, whereas Experiment 2b included the conditions single-context/single-target/2loc, single-context/dual-target/2loc, dual-context/single-target/2loc, and dual-context/dual-target/2loc. This design ensured that, in both experiments, relevant digits appeared as frequently in unique locations as they did in a shared location. Moreover, in Experiment 2b, the number of targets was orthogonal to the number of cued locations, meaning that both single-context (one cued location) and dual context (two cued locations) trials were equally likely to involve one or two relevant items. Another difference was the specific timing of stimulus presentation: whereas Experiments 1a and 1b used a fixed 750 ms inter-stimulus interval within each sequence, Experiments 2a and 2b used a longer, variable interval ranging from 1,000 to 1,500 ms.

### Results and discussion

#### Readiness RT

**Figure 4:**
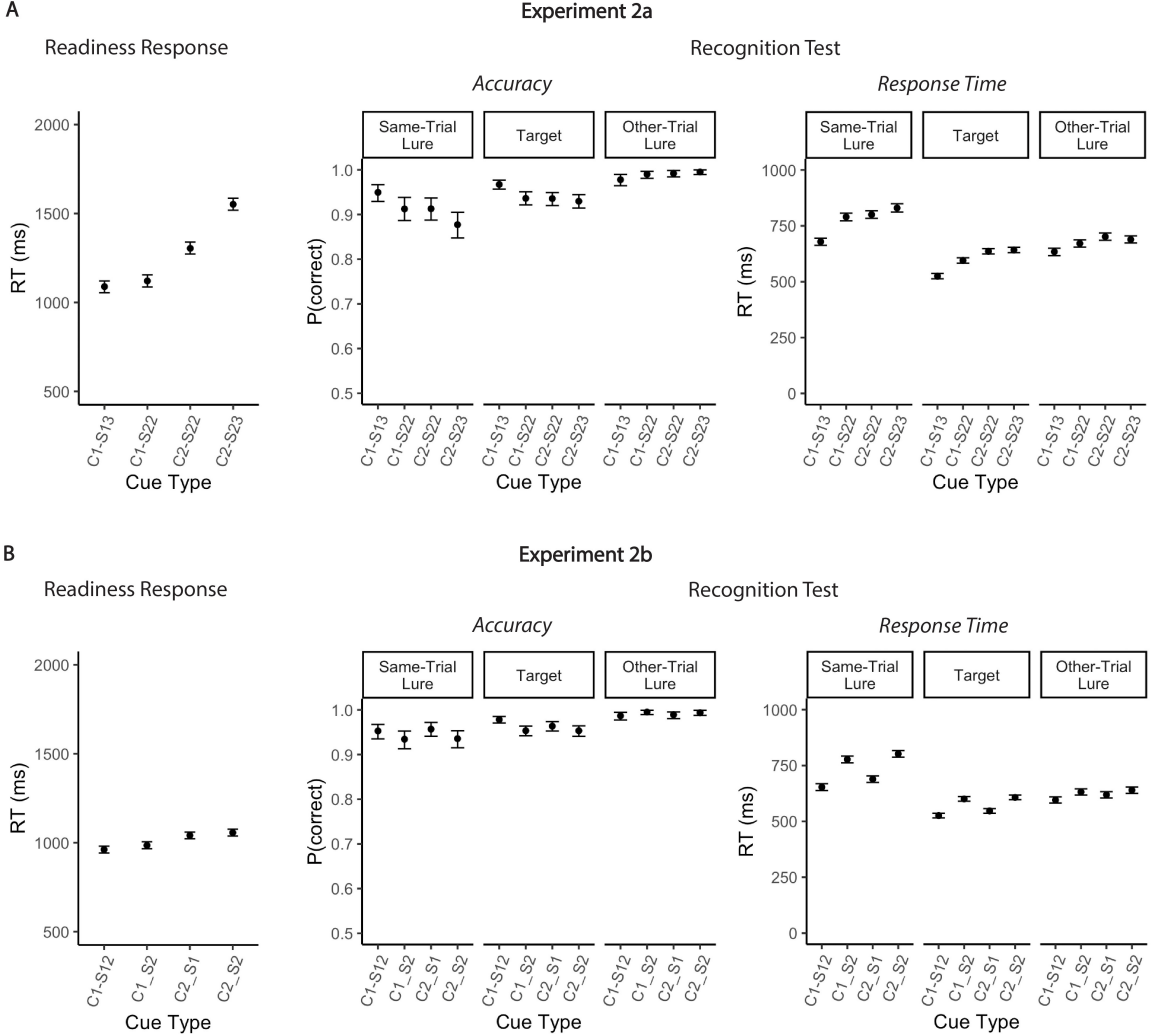
Mean accuracy rates and reaction times for Experiments 2a and 2b. **(A)** Mean response times to readiness prompt **(left)** and mean accuracies and responses times for the recognition test **(right)** in Experiment 2a. **(B)** Mean response times to readiness prompt **(left)** and mean accuracies and responses times for the recognition test **(right)** in Experiment 2b. Error bars represent 95% within-subject confidence intervals. C1, single context; C2, dual context; S1, single-target; S2, dual-target.

Model comparisons confirmed that the full model fit significantly better than the intercept-only model [Experiment 2a: χ^2^(3) = 450.23, *p* < 0.001, Δ AIC = 445, Δ BIC = 424; Experiment 2b: χ^2^(3) = 62.09, *p* < 0.001, Δ AIC = 56, Δ BIC = 35] (Table 3a). Overall, readiness RTs were slower when two locations were cued (dual context) than when only one location was cued (single-context), replicating the spatial context effect observed in Experiments 1a and 1b (Figure 4A). Specifically, in Experiment 2a, response speed was reduced in the dual-context/dual-target/2loc condition compared to the single-context/dual-target/2loc condition [mean difference 184 ms, 95% CI [126, 241], *t*_(7675.2)_ = 7.60, *p* < 0.001; note that due to exclusion of the dual-context/single-target/2loc condition, the effect of context could not be tested for single target trials]. A similar pattern was observed in Experiment 2b, with longer response latencies for dual context trials than for single-context trials, both when one item was relevant [dual-context/single-target/2loc vs. single-context/single-target/2loc: mean difference 80 ms, 95% CI [45, 114], *t*_(8685.1)_ = 5.71, *p* < 0.001] and when two items were relevant [single-context/dual-target/2loc vs. dual-context/dual-target/2loc: mean difference 72 ms, 95% CI [36, 107], *t*_(8685.1)_ = 5.07, *p* < 0.001]. The strong convergence of findings across three experiments indicates that the context effect on readiness RT is quite robust and not a mere artifact of learned task contingencies.

**Table 3a:** Model fit and *post-hoc* comparisons for readiness prompt (Experiments 2a and 2b).

**Experiment 2a**					
Full model: AIC = 124,241, BIC = 124,283, *$R_{adj}^{2}$* = 0.282					
Intercept-only model: AIC = 124,686, BIC = 124,283, *$R_{adj}^{2}$* = 0.241					
Model comparison: *χ^2^(3)* = 450.23, *p* < 0.001					
**Contrast**	**Estimated mean difference**	***df***	***t*** **-value**	***p*** **-value**	**95% CI**
C1-S13 vs. C1-S22	−32	7675.3	−1.32	0.46	[−89, 26]
C1-S22 vs. C2-S22	−184	7675.2	−7.60	< 0.001	[−241, −126]
C2-S22 vs. C2-S23	−247	7675.3	−10.20	< 0.001	[−305, −189]
**Experiment 2b**					
Full model: AIC = 132,238, BIC = 132,280, *$R_{adj}^{2}$* = 0.409					
Intercept-only model: AIC = 132,294, BIC = 132,315, *$R_{adj}^{2}$* = 0.405					
Model comparison: *χ^2^(3)* = 62.09, *p* < 0.001					
**Contrast**	**Estimated mean difference**	***df***	***t*** **-value**	***p*** **-value**	**95% CI**
C1-S12 vs. C1-S22	−24	8685.1	−1.69	0.43	[−61, 13]
C1-S12 vs. C2-S12	−80	8685.1	−5.71	< 0.001	[−116, −43]
C2-S12 vs. C2-S22	−16	8685.1	−1.12	0.70	[−51, 19]
C1-S22 vs. C2-S22	−72	8685.1	−5.07	< 0.001	[−109, −34]
(C1-S12 - C1-S22) vs. (C2-S12 - C2-S22)	−8	8685.1	−0.40	1.00	[−60, 44]
(Cx-S12 - Cx-S22) vs. (C1-Sx - C2-Sx)^a^	−56	8685.1	−3.98	< 0.001	[−93, −19]

CI, confidence interval; C1, single context; C2, dual context; S1, single-target; S2, dual-target.

^a^ThThis contrast tested whether the effect of relevant set size, averaged across C1 and C2 cues, is different from the effect of number of cued contexts, averaged across both relevant set sizes.

However, when testing the effect of relevant set size while holding the number of cued spatial contexts constant, the pattern of results differed from that observed in Experiment 1a. Neither experiment revealed a reliable cost of selecting two items when a single location was cued [single-context/single-target/3loc vs. single-context/dual-target/2loc: mean difference 32 ms, 95% CI [−26, 89], *t*_(7675.3)_ = 1.32, *p* = 0.46; single-context/single-target/2loc vs. single-context/dual-target/2loc: mean difference 24 ms, 95% CI [−11, 59], *t*_(8685.1)_ = 1.69, *p* = 0.31]. Moreover, in Experiment 2b, we did not find a significant effect of relevant set size on readiness RT when two spatial contexts had been cued [dual-context/single-target/2loc vs. dual-context/dual-target/2loc: mean difference 16 ms, 95% CI [−19, 51], *t*_(8685.1)_ = 1.12, *p* = 0.70]. Accordingly, the effect of context was significantly larger than the effect of relevant set size [mean difference 56 ms, 95% CI [19, 93], *t*_(8685.1)_ = 3.98, *p* < 0.001]. Thus, unlike in Experiment 1a, selecting an additional item from WM did not consistently incur an RT cost. Instead, the speed of selection was primarily constrained by the number of cued locations. More specifically, we found the clearest RT increase when two items had to be retrieved from distinct locations (dual-context/dual-target/3loc) than when both targets shared a single spatial context [and the second cued location was unused; dual-context/dual-target/2loc: mean difference 247 ms, 95% CI [189, 305], *t*_(7675.3)_ = 10.20, *p* < 0.001].

Importantly, a comparison of readiness RT patterns across experiments reveals that the conditions in which two targets shared a single spatial context (single-context/dual-target/2loc and dual-context/dual-target/2loc) elicited disproportionally slower responses in Experiments 1a and 1b than in Experiments 2a and 2b. As we have discussed before, a plausible explanation for this discrepancy lies in the relative frequency of single-context/dual-target/2loc and dual-context/dual-target/2loc trials. In Experiments 2a and 2b, single-context/dual-target/2loc and dual-context/dual-target/2loc conditions accounted for 50% of trials, whereas in Experiments 1a and 1b, they accounted for only 33% of trials. Consequently, the lower frequency of trials requiring the retrieval of two items from a single location in Experiments 1a and 1b may have contributed to slower RTs in those conditions. By contrast, the results of Experiment 2b indicate that the second potential confound—mismatch between number of cued spatial contexts and number of targets—is unlikely account for the performance differences between dual context and single-context conditions: although there is a mismatch in both single-context/dual-target/2loc and dual-context/single-target/2loc conditions, readiness RTs were substantially faster for the former (single-context) than the latter (dual context), adding further credibility to the notion that greater context competition slows down selection from WM.

#### Recognition test

Accuracy rates and RTs for probe responses were consistent across Experiments 2a and 2b and largely replicated the results from Experiment 1a (Figure 4B, Tables 3b, c). Notably, errors rates were again slightly higher when two targets had to be retrieved from a single location (single-context/dual-target/2loc) compared to when only one target was retrieved from that location (single-context/single-target/2loc), indicating that participants occasionally failed to access the second relevant item in the single-context/dual-target/2loc condition. In addition, probe RTs were slower when two spatial contexts were cued (dual context) rather than only one (single-context). This effect, which mirrors the findings from Experiment 1a, may reflect that the prolonged and/or noisier selection process on dual context trials (as evidenced by slower readiness responses) allowed additional evidence to accumulate for irrelevant items. Lingering activation of these irrelevant items may have delayed the subsequent recognition decision, particularly when the probe was a target or a same-trial lure and therefore could not be quickly dismissed based on item memory alone.

**Table 3b:** Model fit and *post-hoc* comparisons for probe response (Experiment 2a).

**Accuracy**				
Full model: AIC = 3405, BIC = 3496, *$R_{adj}^{2}$* = 0.267				
Reduced model (both main effects): AIC = 3413, BIC = 3462, *$R_{adj}^{2}$* = 0.242				
Reduced model (main effect probe type only): AIC = 3434, BIC = 3461, *$R_{adj}^{2}$* = 0.228				
Reduced model (main effect selection demand only): AIC = 3569, BIC = 3604, *$R_{adj}^{2}$* = 0.100				
Model comparison (full model vs. no interaction): *χ^2^(6)* = 20.17, *p* = 0.003				
Model comparison (both main effects vs. probe type only): *χ^2^(3)* = 26.57, *p* < 0.001				
Model comparison (both main effects vs. selection demand only): *χ^2^(2)* = 160.50, *p* < 0.001				
**Contrast**	**Odds ratio**	***z*** **-value**	***p*** **-value**	**95% CI**
Same-trial lure vs. other-trial lure	0.11	−9.50	< 0.001	[0.05, 0.22]
Target vs. other-trial lure	0.17	−7.78	< 0.001	[0.09, 0.33]
Same-trial lure vs. target	0.65	−4.23	< 0.001	[0.48, 0.87]
C1-S13 vs. C1-S22	1.18	0.87	0.17	[0.67, 2.11]
C1-S22 vs. C2-S22	0.94	−0.26	1.00	[0.49, 1.81]
C2-S22 vs. C2-S23	0.99	−0.04	1.00	[0.47, 2.10]
Same-trial lure: C1-S13 vs. C1-S22	1.85	2.49	0.17	[0.90, 3.80]
Other-trial lure: C1-S13 vs. C1-S22	0.45	−1.62	0.81	[0.11, 1.91]
Target: C1-S13 vs. C1-S22	2.01	3.43	0.009	[1.11, 3.64]
Same-trial lure: C1-S22 vs. C2-S22	0.99	−0.03	1.00	[0.53, 1.87]
Other-trial lure: C1-S22 vs. C2-S22	0.85	−0.26	1.00	[0.15, 5.00]
Target: C1-S22 vs. C2-S22	0.99	−0.04	1.00	[0.60, 1.64]
Same-trial lure: C2-S22 vs. C2-S23	1.47	1.90	0.59	[0.81, 2.67]
Other-trial lure: C2-S22 vs. C2-S23	0.59	−0.73	1.00	[0.07, 4.91]
Target: C2-S22 vs. C2-S23	1.12	0.68	1.00	[0.69, 1.84]
**RT**					
Full model: AIC = 102,894, BIC = 102,991, *$R_{adj}^{2}$* = 0.439					
Reduced model (both main effects): AIC = 102,922, BIC = 102,977, *$R_{adj}^{2}$* = 0.436					
Reduced model (main effect probe type only): AIC = 103,321, BIC = 103,356, *$R_{adj}^{2}$* = 0.405					
Reduced model (main effect selection demand only): AIC = 103,926, BIC = 103,968, *$R_{adj}^{2}$* = 0.355					
Model comparison (full model vs. no interaction): *χ^2^(6)* = 40.05, *p* < 0.001					
Model comparison (both main effects vs. probe type only): *χ^2^(3)* = 405.32, *p* < 0.001					
Model comparison (both main effects vs. selection demand only): *χ^2^(2)* = 1,008.30, *p* < 0.001					
**Contrast**	**Estimated mean difference**	***df***	***t*** **-value**	***p*** **-value**	**95% CI**
Same-trial lure vs. other-trial lure	101	7683.4	16.70	< 0.001	[83, 119]
Target vs. other-trial lure	−74	7683.1	−14.57	< 0.001	[−89, −60]
Same-trial lure vs. target	175	7683.4	32.79	< 0.001	[160, 191]
C1-S13 vs. C1-S22	−73	7683.2	−11.47	< 0.001	[−91, −54]
C1-S22 vs. C2-S22	−27	7683.1	−4.31	< 0.001	[−46, −9]
C2-S22 vs. C2-S23	−7	7683.2	−1.17	0.98	[−26, 11]
Same-trial lure: C1-S13 vs. C1-S22	−111	7683.2	−9.00	< 0.001	[−147, −75]
Other-trial lure: C1-S13 vs. C1-S22	−37	7683.1	−3.16	0.024	[−71, −3]
Target: C1-S13 vs. C1-S22	−70	7683.2	−8.24	< 0.001	[−95, −45]
Same-trial lure: C1-S22 vs. C2-S22	−10	7683.2	−0.84	1.00	[−47, 26]
Other-trial lure: C1-S22 vs. C2-S22	−31	7683.1	−2.62	0.12	[−65, 4]
Target: C1-S22 vs. C2-S22	−41	7683.1	−4.82	< 0.001	[−67, −16]
Same-trial lure: C2-S22 vs. C2-S23	−29	7683.3	−2.32	0.27	[−66, 8]
Other-trial lure: C2-S22 vs. C2-S23	13	7683.1	1.11	0.99	[−21, 47]
Target: C2-S22 vs. C2-S23	−6	7683.2	−0.70	1.00	[−31, 19]

CI, confidence interval; C1, single context; C2, dual context; S1, single-target; S2, dual-target.

**Table 3c:** Model fit and *post-hoc* comparisons for probe response (Experiment 2b).

**Accuracy**				
Full model: AIC = 3,101, BIC = 3,194, *$R_{adj}^{2}$* = 0.305				
Reduced model (both main effects): AIC = 3,104, BIC = 3,154, *$R_{adj}^{2}$* = 0.290				
Reduced model (main effect probe type only): AIC = 3,111, BIC = 3,139, *$R_{adj}^{2}$* = 0.284				
Reduced model (main effect selection demand only): AIC = 3,216, BIC = 3,251, *$R_{adj}^{2}$* = 0.199				
Model comparison (full model vs. no interaction): *χ^2^(6)* = 14.25, *p* = 0.027				
Model comparison (both main effects vs. probe type only): *χ^2^(3)* = 13.20, *p* = 0.004				
Model comparison (both main effects vs. selection demand only): *χ^2^(2)* = 116.06, *p* < 0.001				
**Contrast**	**Odds ratio**	***z*** **-value**	***p*** **-value**	**95% CI**
Same-trial lure vs. other-trial lure	0.15	−8.85	< 0.001	[0.08, 0.28]
Target vs. other-trial lure	0.22	−7.09	< 0.001	[0.12, 0.42]
Same-trial lure vs. target	0.66	−3.81	0.003	[0.47, 0.91]
C1-S12 vs. C1-S22	1.04	0.18	1.00	[0.54, 2.01]
C1-S12 vs. C2-S12	1.09	0.44	1.00	[0.62, 1.90]
C2-S12 vs. C2-S22	1.02	0.11	1.00	[0.54, 1.92]
C1-S22 vs. C2-S22	1.07	0.27	1.00	[0.52, 2.20]
Same-trial lure: C1-S12 vs. C1-S22	1.41	1.45	0.95	[0.69, 2.85]
Other-trial lure: C1-S12 vs. C1-S22	0.37	−1.73	0.81	[0.06, 2.08]
Target: C1-S12 vs. C1-S22	2.19	3.68	0.004	[1.16, 4.14]
Same-trial lure: C1-S12 vs. C2-S12	0.90	−0.42	1.00	[0.42, 1.93]
Other-trial lure: C1-S12 vs. C2-S12	0.83	−0.40	1.00	[0.22, 3.21]
Target: C1-S12 vs. C2-S12	1.71	2.42	0.26	[0.88, 3.32]
Same-trial lure: C2-S12 vs. C2-S22	1.51	1.73	0.81	[0.74, 3.12]
Other-trial lure: C2-S12 vs. C2-S22	0.55	−1.07	1.00	[0.10, 2.91]
Target: C2-S12 vs. C2-S22	1.28	1.33	0.98	[0.73, 2.23]
Same-trial lure: C1-S22 vs. C2-S22	0.97	−0.14	1.00	[0.50, 1.88]
Other-trial lure: C1-S22 vs. C2-S22	1.26	0.35	1.00	[0.17, 9.22]
Target: C1-S22 vs. C2-S22	1.00	−0.01	1.00	[0.59, 1.69]
**RT**					
Full model: AIC = 115,102, BIC = 115,201, *$R_{adj}^{2}$* = 0.379					
Reduced model (both main effects): AIC = 115,167, BIC = 115,223, *$R_{adj}^{2}$* = 0.374					
Reduced model (main effect probe type only): AIC = 115,526, BIC = 115,562, *$R_{adj}^{2}$* = 0.347					
Reduced model (main effect selection demand only): AIC = 116,266, BIC = 116,309, *$R_{adj}^{2}$* = 0.287					
Model comparison (full model vs. no interaction): *χ^2^(6)* = 76.55, *p* < 0.001					
Model comparison (both main effects vs. probe type only): *χ^2^(3)* = 365.89, *p* < 0.001					
Model comparison (both main effects vs. selection demand only): *χ^2^(2)* = 1103.60, *p* < 0.001					
**Contrast**	**Estimated mean difference**	***df***	***t*** **-value**	***p*** **-value**	**95% CI**
Same-trial lure vs. other-trial lure	109	8693.5	20.71	< 0.001	[93, 125]
Target vs. other-trial lure	−51	8693.1	−11.45	< 0.001	[−65, −38]
Same-trial lure vs. target	160	8693.6	34.57	< 0.001	[146, 174]
C1-S12 vs. C1-S22	−78	8693.2	−14.10	< 0.001	[−95, −62]
C1-S12 vs. C2-S12	−27	8693.2	−4.83	< 0.001	[−43, −10]
C2-S12 vs. C2-S22	−64	8693.2	−11.71	< 0.001	[−82, −48]
C1-S22 vs. C2-S22	−13	8693.2	−2.39	0.28	[−30, 3]
Same-trial lure: C1-S12 vs. C1-S22	−124	8693.5	−11.46	< 0.001	[−156, −91]
Other-trial lure: C1-S12 vs. C1-S22	−36	8693.1	−3.52	0.008	[−67, −5]
Target: C1-S12 vs. C1-S22	−75	8693.1	−10.05	< 0.001	[−97, −53]
Same-trial lure: C1-S12 vs. C2-S12	−36	8693.2	−3.34	0.016	[−67, −4]
Other-trial lure: C1-S12 vs. C2-S12	−23	8693.1	−2.29	0.35	[−54, 7]
Target: C1-S12 vs. C2-S12	−21	8693.2	−2.81	0.090	[−43, 1]
Same-trial lure: C2-S12 vs. C2-S22	−113	8693.2	−10.54	< 0.001	[−145, −81]
Other-trial lure: C2-S12 vs. C2-S22	−21	8693.1	−2.04	0.55	[−52, 10]
Target: C2-S12 vs. C2-S22	−61	8693.2	−8.11	< 0.001	[−80, −38]
Same-trial lure: C1-S22 vs. C2-S22	−25	8693.2	−2.27	0.36	[−58, 8]
Other-trial lure: C1-S22 vs. C2-S22	−8	8693.1	−0.81	1.00	[−39, 23]
Target: C1-S22 vs. C2-S22	−7	8693.2	−0.92	1.00	[−29, 16]

CI, confidence interval; C1, single context; C2, dual context; S1, single-target; S2, dual-target.

## Experiment 2c

As outlined above, the results of Experiments 2a and 2b suggest that the relative unexpectedness of trials in which two targets occupied the same spatial location may have affected the performance on single-context/dual-target/2loc and dual-context/dual-target/2loc trials in Experiments 1a and 1b. To further test this hypothesis and validate the findings from Experiments 2a and 2b, we conducted an additional experiment. The follow-up task replicated the design of Experiment 1a but increased the number of single-context/dual-target/2loc and dual-context/dual-target/2loc trials. Consequently, trials in which both targets were bound to a single spatial context (single-context/dual-target/2loc, dual-context/dual-target/2loc) and trials in which the targets appeared in distinct locations (single-context/single-target/3loc, single-context/single-target/2loc, dual-context/single-target/2loc, dual-context/dual-target/3loc) were equally frequent, each accounting for 50% of the total number of trials. Thus, if the lower frequency of dual target2 trials in Experiment 1a contributed to increased readiness RTs in the corresponding conditions, then this frequency-based bias should be reduced, or even eliminated, in the current design. In that case, the resulting performance pattern should resemble that of Experiments 2a and 2b—namely, readiness RTs should increase with the number of cued locations rather than the number of selected items.

### Methods

#### Participants

Fifty-two young adults were recruited from an introductory psychology course at Queens College and received course credit for their participation. Twenty participants were excluded from data analyses due to low accuracy (*n* = 16) or exceedingly slow responses (*n* = 4). The final sample thus included 32 datasets (mean age 25.9 years, *SD* = 7.54, range 18–40 years; 17 females, 14 males, one non-binary/other; see Supplementary methods for racial and ethnic composition).

#### Design, stimuli, and procedure

All stimuli and procedures were identical to those in Experiment 1a, except that the single-context/dual-target/2loc and dual-context/dual-target/2loc conditions included twice as many trials (96 each) as the single-context/single-target/3loc, single-context/single-target/2loc, dual-context/single-target/2loc, and dual-context/dual-target/3loc conditions (48 each).

### Results and discussion

**Figure 5:**
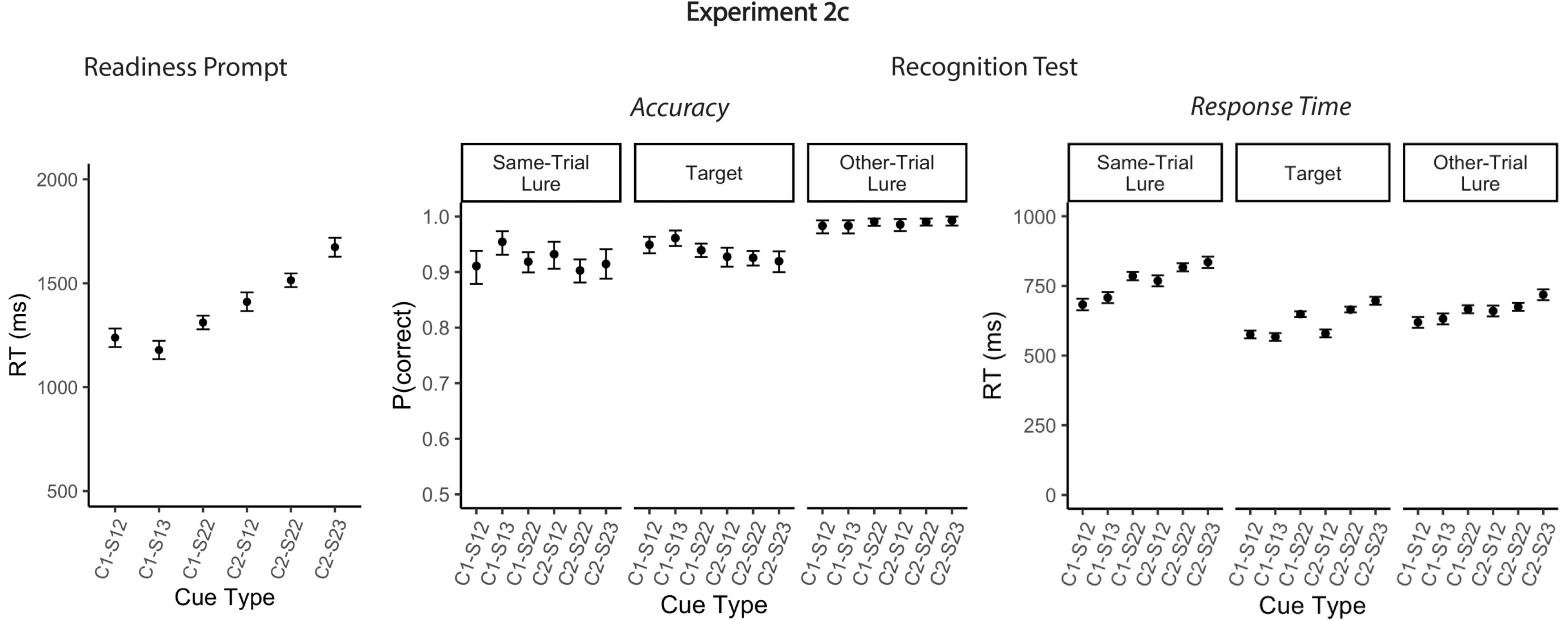
Mean accuracy rates and reaction times for Experiments 2c. Mean response times to readiness prompt **(Left)** and mean accuracies and responses times for the recognition test **(Right)** in Experiment 2c. Error bars represent 95% within-subject confidence intervals. C1, single context; C2, dual context; S1, single-target; S2, dual-target.

The results for the probe response were consistent with the preceding experiments and will therefore not be further discussed (the interested reader may refer to Figure single target, Table single target). In line with the results of the previous experiments, Experiment 2c revealed a robust effect of cue type on readiness RTs. Responses were significantly slower when two locations were cued than when one locations was cued, for both single-target trials [single-context/single-target/2loc vs. dual-context/single-target/2loc: mean difference 173 ms, 95% CI [84, 263], *t*_(10992.1)_ = 5.28, *p* < 0.001] and dual-target trials [single-context/dual-target/2loc vs. dual-context/dual-target/2loc: mean difference 205 ms, 95% CI [141, 269], *t*_(10992.0)_ = 8.79, *p* < 0.001; Table 4, Figure 5]. In contrast, the effect of relevant set size—i.e., the number of target items—was substantially smaller than in Experiment 1a. Like in Experiments 2a and 2b, readiness RTs did not differ significantly single- and dual-target trials when one location was cued [single-context/single-target/2loc vs. single-context/dual-target/2loc: mean difference 72 ms, 95% CI [−5, 150], *t*_(10992.1)_ = 2.54, *p* = 0.086]. However, when two locations were cued, RTs were slower for dual-target compared to single-target trials [dual-context/single-target/2loc vs. dual-context/dual-target/2loc: mean difference 104 ms, 95% CI [26, 182], *t*_(10992.1)_ = 3.65, *p* = 0.002]. Overall, the effect of relevant set size remained significantly smaller than the effect of cue type [mean difference 101 ms, 95% CI [24, 179], *t*_(10992.1)_ = 3.56, *p* = 0.003]. As in Experiments 1a and 1b, RTs did not vary depending on whether digits appeared in two or three distinct locations when only one item was relevant [single-context/single-target/2loc vs. single-context/single-target/3loc: mean difference 60 ms, 95% CI [−29, 149], *t*_(10992.1)_ = 1.83, *p* = 0.43]. In contrast, on dual-target trials, responses were slower in the dual-context/dual-target/3loc compared to the dual-context/dual-target/2loc conditions [mean difference 159 ms, 95% CI [80, 236], *t*_(10992.1)_ = 5.54, *p* < 0.001]. Taken together, these findings suggest that the effect of relevant set size observed in Experiment 1a was likely inflated by the lower expectancy of single-context/dual-target/2loc and dual-context/dual-target/2loc trials in which two targets shared a single location. The current results thus further substantiate the conclusion that the speed of selection from WM is more strongly constrained by competition at the context level than at the item level.

**Table 4:** Model fit and *post-hoc* comparisons for readiness prompt (Experiment 2c).

Full model: AIC = 180,408, BIC = 180,466, ***R$R_{adj}^{2}$*** = 0.270					
Intercept-only model: AIC = 180,750, BIC = 180,772, *$R_{adj}^{2}$* = 0.246					
Model comparison: *χ^2^(5)* = 352.39, *p* < 0.001					
**Contrast**	**Estimated mean difference**	***df***	***t*** **-value**	***p*** **-value**	**95% CI**
C1-S12 vs. C1-S13	60	10992.1	1.83	0.43	[−29, 149]
C1-S12 vs. C1-S22	−72	10992.1	−2.54	0.086	[−150, 5]
C1-S12 vs. C2-S12	−173	10992.1	−5.28	< 0.001	[−263, −84]
C1-S22 vs. C2-S22	−205	10992.0	−8.79	< 0.001	[-269,−141]
C2-S12 vs. C2-S22	−104	10992.1	−3.65	0.002	[−182, −26]
C2-S22 vs. C2-S23	−158	10992.1	−5.54	< 0.001	[−236, −80]
(C1-S12 to C1-S22) vs. (C2-S12 to C2-S22)	32	10992.1	0.79	0.99	[−78, 141]
(Cx-S12 to Cx-S22) vs. (C1-Sx to C2-Sx)^a^	−101	10992.1	−3.56	0.003	[−179, −24]

CI, confidence interval; C1, single context; C2, dual context; S1, single-target; S2, dual-target.

^a^ThThis contrast tested whether the effect of relevant set size, averaged across C1 and C2 cues, is different from the effect of number of cued contexts, averaged across both relevant set sizes.

## General discussion

Simultaneously prioritizing multiple WM contents for use in thought and action takes time and is prone to error. In the present study, we asked whether this multi-selection performance cost arises primarily from competition between the spatial context representations that guide WM access, from competition between the memory items themselves, or from both. To distinguish between these possibilities, we used a spatial retrocuing paradigm in which we independently manipulated (1) the number of cued locations (i.e., spatial contexts) and (2) the number of to-be-selected memory items. Across five experiments, we consistently found that cueing two spatial locations—compared to one—substantially delayed selection, even when the number of relevant items was held constant. By contrast, relevant set size exerted a weaker and less consistent influence on selection speed. Notably, effects on accuracy were generally small, suggesting that the number of concurrently cued spatial contexts and items affected the efficiency, rather than the fidelity, of WM selection.

### Context competition slows down the selection of multiple items from working memory

The robust effect of the number of cued spatial locations on selection speed confirms and extends findings from our prior study in which each memory item was bound to a distinct spatial context (Tiferet-Dweck et al., 2025). The current results suggest that the cost of prioritizing multiple WM items cannot be fully explained by competitive interactions at either the context or item level alone. Rather, both factors contribute to the increased cognitive demands of multi-item retrieval, with context-level competition exerting the stronger influence. At the item-level, previous work has shown that increasing WM load weakens the neural response associated with individual items, reducing their discriminability (Bays, 2015; Carandini and Heeger, 2011; Sprague et al., 2014). A similar mechanism may be at work at the context level: when two spatial contexts are active simultaneously, competitive interference may weaken their neural representations. This mutual degradation reduces the capacity of each context to effectively bias item selection, thereby slowing evidence accumulation and increasing selection time. In contrast, when only a single spatial context is cued, it can exert a stronger, undiluted influence on downstream item selection, facilitating faster retrieval. This notion is consistent with the assumptions of the WM gating framework which assumes competitive relationships among representations across all levels of the representational hierarchy, including higher-order contexts (Badre and Frank, 2012; Chatham and Badre, 2015; Frank and Badre, 2012).

Another, not mutually exclusive possibility is that a shared spatial context allows faster access because the corresponding items become integrated in memory. Such integration of overlapping memories has been described for both WM and LTM processes and may rely on autoassociative pattern completion, i.e., the retrieval of a complete memory—consisting of several elements that have been associated with each other at encoding—based on partial input (Manohar et al., 2019; Schlichting and Preston, 2015). In the present WM paradigm, memory integration could occur when a new digit is presented in a grid location that had been used earlier in the same sequence. Re-using the same location may trigger re-activation of the digit that had appeared there before. As a result, both current and previous digit might be bound not only to their shared spatial context but also directly to each other. During retrieval, cueing the joint location will not merely reactivate both target items individually, but the two items will also serve as retrieval cues for each other, leading to their mutual enhancement. By contrast, if the target items are bound to distinct spatial locations, cueing these locations will trigger re-activation of each item through their individual spatial context, but they will not directly activate each other. Consequently, evidence accumulation for targets with distinct spatial contexts may be slower than for targets that share a context.

Despite the potential benefits on retrieval efficiency, sharing the same spatial context may also have detrimental effects on multi-item selection. For instance, the interference model posits that increased context similarity leads to simultaneous activation of multiple items, which may result in their mutual distortion (Oberauer and Lin, 2017, 2023). By implication, if several items share the same spatial context, they may be more easily confused or blended. Similar ideas of representational interference are integral to many contemporary WM models (e.g., Buschman, 2021; Manohar et al., 2019). It may therefore seem contradictory that we did not observe any clear accuracy differences between conditions in which two target items were bound to distinct spatial contexts (dual context) vs. a single joint context (single-context). We can think of two factors that might account for the absence of such differences: First, since the items that shared a context were both targets, confusion errors could not occur. Second, the discrete nature of the memory contents themselves may have made them less vulnerable to blending. Since the representations of individual digits are likely well-separated in neural space, they might be less affected by representational interference (cf. Buschman, 2021).

By revealing a possible source of limitations in multi-item WM retrieval, our findings also offer insights into the question of whether the focus of attention can hold only one or several elements (Oberauer and Bialkova, 2009, 2011; Gilchrist and Cowan, 2011). While previous studies have focused primarily on the number of items, the present results suggest that the true capacity limit of the focus of attention is linked to the number of simultaneously active spatial context representations. It is possible that the focus can hold only one context at a time but can accommodate multiple items if they are all bound to this same context. The idea that multiple items can be simultaneously kept in an active state in the focus of attention when they are bound to a joint group context has previously been advanced by (Oberauer 2018). In contrast, distinct contexts may need to be retrieved sequentially into the focus, resulting in a delay if item selection depends on more than one context. Such a scenario would be consistent with our previous finding that retrieval of two WM items cannot be fully parallel if each item is accessed through its unique spatial location Tiferet-Dweck et al., (2025). Access speed thus might be primarily constrained by the question of how many “handles” are needed to retrieve contents from WM, regardless of whether one or several items are attached to the same handle.

### Potential confounds and alternative accounts

Importantly, the cost associated with cueing multiple spatial contexts, as observed here, did not merely reflect a speed-accuracy trade-off. When accuracy effects did emerge, they were small and went in the same direction as the RT effects, i.e., performance declined as the number of relevant contexts and/or items increased. This observation is consistent with our prior finding that response accuracy decreases with larger relevant set sizes under conditions in which each item is bound to a unique spatial context (Tiferet-Dweck et al., 2025). As in our previous work, this effect was specific to binding memory: error rates increased for intrusion and target probes, but remained largely unaffected when participants could rely on item memory alone (other-trial lures).

Still, one might expect higher error rates for expect dual-target trials than single-target trials for mere probabilistic reasons: Item selection is a stochastic process, with the probability of errors increasing as more items need to be retrieved. However, owing to the small set size of three memory items, in the current study, the probability of guessing the correct set of items(s)—whether it included one or two digits—was equal across single- and dual-target conditions (one-third). Moreover, WM load likely remained within the capacity limits of most participants. This allowed them to maintain item-context bindings with relatively high fidelity. Thus, while context- and item-level competition may have slowed evidence accumulation, it did not appear to degrade WM representations enough to cause substantial forgetting.

Another potential concern for the interpretation of the context-related RT effect in the present study is that when unused spatial locations were cued, as was the case in the dual-context/single-target/2loc and dual-context/dual-target/2loc conditions, responses may have been prolonged because participants were simply confused. However, as we have discussed above, the results for the dual-context/dual-target/3loc condition (Experiments 2a and 2c), in which both cued locations contained a digit, contradict this account. Readiness responses on dual-context/dual-target/3loc trials were slower than those on dual-context/dual-target/2loc trials, despite both conditions involving the same number of cued spatial contexts and relevant items. Importantly, the RT difference between dual-context/dual-target/2loc and dual-context/dual-target/3loc conditions cannot merely be attributed to the fact that items appeared in two vs. three distinct locations as the comparison of single-context/single-target/2loc and single-context/single-target/3loc conditions consistently failed to reveal a comparable difference. A more plausible explanation for the faster readiness responses in the dual-context/dual-target/2loc condition is that participants could rely on item memory to quickly discern that one of the cued locations had not been used in the current trial, allowing them to abandon any item retrieval attempts for that location.

Finally, it is important to note that readiness RTs might not provide a fully unbiased estimate of WM access speed. Experiment 1b, which omitted the readiness response and offered a more objective measure of selection speed, replicated the context-specific effects, thereby supporting a central conclusion of the present study. Nonetheless, readiness RTs may have underestimated item-number effects. This could occur if participants tended to respond prematurely on dual-target trials, before completing item selection, or if they delayed responses on single-target trials. The first possibility should have led to reduced recognition accuracy on dual-target relative to single-target trials, which was not observed. The second possibility—additional monitoring on single-target trials—appears more plausible. For example, participants may have double-checked that no further item needed to be selected. Future studies could employ more sensitive neural measures, such as EEG, to more precisely quantify the relative contributions of item- vs. context-level competition to limitations in multi-item WM access.

### Limitations to generalizability

While relatively high exclusion rates due to poor performance are not unusual for online experiments, they raise the possibility that the present findings may not generalize to lower-performing subpopulations. However, it is important to note that the overall pattern of results remained qualitatively unchanged when data from all participants were included. Furthermore, the core findings were similar across samples drawn from a college subject pool (Experiments 1a and 2a) and from the general U.S. population (Experiments 1b, 2b, and 2c), despite variations in age, gender, race, or ethnicity. We therefore believe the main effects are reproducible in samples of healthy adults within Western cultural contexts.

A more open question is whether the present findings extend to WM for non-verbal, continuous features and non-sequentially presented material. Despite this uncertainty, recent theoretical and empirical work indicates common mechanisms underlying WM for both discrete, sequential lists and simultaneously presented visuospatial information (see Oberauer and Lin, 2023). Nonetheless, we consider it likely that WM tasks requiring the reproduction of continuous features (e.g., color or orientation) are more sensitive to precision loss, confusion, or blending of memory representations in multi-item retrieval. These effects may be particularly pronounced when using larger set sizes that exceed typical WM capacity limits. Moreover, it remains unclear whether the present findings generalize to scenarios in which non-spatial features serve as context. Both theoretical and empirical work suggest that context competition effects are unlikely to depend on the *type* of context *per se*, but rather on the degree of representational overlap between the item-specific context representations (Oberauer and Lin, 2017, 2023). Supporting this view, unpublished data from our laboratory indicate that performance cost of multi-item WM selection are highly consistent across spatial and non-spatial contexts, including color, shape, or symbolic context cues.

### Conclusions and future directions

In this study, we demonstrated that the performance cost associated with selecting multiple items from WM is more strongly linked to the number of contexts guiding the selection process than to the number of relevant items. However, these two factors were not completely independent: context-related costs were larger when two items, rather than only one, had to be prioritized. This suggests that the major bottleneck arises not simply from the need to represent or reactivate multiple contexts, but from actually *using* those context representations to retrieve the relevant items. While our findings have important implications for theories of WM selection by specifying boundary conditions of multi-item access, they leave open the question of whether the observed bottleneck reflects limitations in stimulus-driven (bottom-up) processing or in top-down control mechanisms. When multiple contexts are cued simultaneously, control demands likely increase as greater representational overlap at the context level intensifies competition and interference. As a result, stronger top-down biasing may be required to disambiguate context cues and may force item to proceed serially rather than fully in parallel. If, as is widely assumed, higher-order control signals are abstract and content-free, they should be less susceptible to sensory-level overlap than bottom-up, cue-driven inputs. One plausible neurocognitive mechanism for implementing such top-down influences on WM selection is hierarchical corticostriatal gating, whereby higher-order context representations regulate access to relevant lower-order WM representations. Other accounts have proposed that abstract control is mediated by content-independent pointers that allow spatiotemporal tracking of WM contents (Awh and Vogel, 2025), or by low-dimensional spatiotemporal patterns of alpha/beta synchrony in PFC (Buschmann and Miller, 2022). Future studies may use neuroimaging and/or electrophysiological techniques to offer a more complete picture of how stimulus-driven and top-down processes impose limits on multi-item selection—and why these limits may be more pronounced at the context level. For instance, new insights could be gained by comparing the neural decoding of context-specific and item-specific information for single- vs. dual-context cue conditions, and by examining whether such information emerges first in higher-order control regions before propagating to lower-order sensory regions—consistent with a top-down biasing account.

## Data Availability

The datasets presented in this study can be found in online repositories. The names of the repository/repositories and accession number(s) can be found below: https://osf.io/b63jz/?view_only=329137a12c014ba3aa563c47f65141f6.

## Footnotes

1 There is some debate as to whether item-context bindings are temporary or long-lasting (cf. Beukers et al., 2021).
