# Supplementary material for: Limits on selecting multiple items from working memory: the role of context and item competition
Source: Front Cognit. 2025 Nov 11;4:1668316. doi: 10.3389/fcogn.2025.1668316 (PMC13281070; doi:10.3389/fcogn.2025.1668316)
Supplement: Supplementary file 1 [file Table_1.docx]

**Supplementary Materials**

***Supplementary Methods***

**Experiment 1a.** Forty percent of the participants self-identified as non-Hispanic Asian, 15% as Hispanic Other-Race, 13% as non-Hispanic Black, 8% as Hispanic White, 5% as non-Hispanic White, 5% as non-Hispanic Other-Race, and 3% as Hispanic Asian. One participant self-identified as Asian and preferred not to report their ethnicity. Two participants identified as Hispanic and non-Hispanic, respectively, but did not report their race. Another two participants did not report either race or ethnicity.

**Experiment 1b.** Fifty percent of the participants self-identified as non-Hispanic White, 30% as non-Hispanic Black, 10%, as Hispanic White, 3% as non-Hispanic native Hawaiian and Other Pacific Islander, and 3% as non-Hispanic Asian. Two participants identified as Hispanic but did not report their race.

**Experiment 2a.** Twenty-six percent of the participants self-identified as non-Hispanic Asian, 15% as non-Hispanic White, 13% as Hispanic Other-Race, 11% as Hispanic White, 11 % as Hispanic Black, 9% as non-Hispanic Black, and 2% as non-Hispanic Other-Race. Two participants self-identified as Asian and White, respectively, and preferred not to report their ethnicity. Two participants identified as Hispanic but did not report their race. One participant identified as non-Hispanic and did not report their race. Another participant did not report either race or ethnicity.

**Experiment 2b.** Fifty-nine percent of the participants self-identified as non-Hispanic White, 20% as non-Hispanic Black, 12% as non-Hispanic Asian, 5% as Hispanic White, and 4% as Hispanic Black.

**Experiment 2c.** Thirty-one percent of the participants self-identified as non-Hispanic Black, 28% as non-Hispanic White, 19% as non-Hispanic Asian, 6% as Hispanic White, 6% as Hispanic Other-Race, and 3% as non-Hispanic Native Hawaiian and Other Pacific Islander. Two participants self-identified as Hispanic but did not report their race.

**Table S1**

*Model Fit and Post-Hoc Comparisons for Probe Response (Experiment 2c)*

| ***Accuracy***  Full model: AIC = 5070, BIC = 5210, *R^2^_adj_* = 0.248  Reduced model (both main effects): AIC = 5064, BIC = 5130, *R^2^_adj_* = 0.242  Reduced model (main effect probe type only): AIC = 5076, BIC = 5105, *R^2^_adj_* = 0.234  Reduced model (main effect selection demand only): AIC = 5280, BIC = 5332, *R^2^_adj_* = 0.103  Model comparison (full model vs. no interaction): *χ^2^(10)* = 13.74, *p* = .19  Model comparison (both main effects vs. probe type only): *χ^2^(5)* = 21.81, *p* < .001  Model comparison (both main effects vs. selection demand only): *χ^2^(2)* = 220.36, *p* < .001 | | | | | |
| --- | --- | --- | --- | --- | --- |
| Contrast | Odds Ratio |  | *z*-value | *p*-value | 95% CI |
| Same-Trial Lure vs. Other-Trial Lure | 0.14 |  | -10.51 | <.001 | [0.08, 0.26] |
| Target vs. Other-Trial Lure | 0.18 |  | -9.60 | <.001 | [0.10, 0.31] |
| Same-Trial Lure vs. Target | 0.80 |  | -2.45 | .33 | [0.60, 1.06] |
| C1-S12 vs. C1-S13 | 0.71 |  | -1.57 | .97 | [0.36, 1.40] |
| C1-S12 vs. C1-S22 | 0.85 |  | -0.83 | 1.00 | [0.46, 1.57] |
| C1-S12 vs. C2-S12 | 0.97 |  | -0.14 | 1.00 | [0.49, 1.91] |
| C1-S22 vs. C2-S12 | 1.14 |  | 0.65 | 1.00 | [0.60, 2.16] |
| C1-S22 vs. C2-S22 | 1.15 |  | 0.77 | 1.00 | [0.65, 2.03] |
| C2-S12 vs. C2-S22 | 1.01 |  | 0.03 | 1.00 | [0.53, 1.90] |
| C2-S22 vs. C2-S23 | 0.89 |  | -0.49 | 1.00 | [0.42, 1.89] |
| Same-Trial Lure: C1-S12 vs. C1-S13 | 0.47 |  | -2.53 | .28 | [0.19, 1.19] |
| Other-Trial Lure: C1-S12 vs. C1-S13 | 1.00 |  | -0.01 | 1.00 | [0.19, 5.32] |
| Target: C1-S12 vs. C1-S13 | 0.75 |  | -1.19 | 1.00 | [0.35, 1.59] |
| Same-Trial Lure: C1-S12 vs. C1-S22 | 0.89 |  | -0.53 | 1.00 | [0.45, 1.77] |
| Other-Trial Lure: C1-S12 vs. C1-S22 | 0.57 |  | -1.09 | 1.00 | [0.11, 2.87] |
| Target: C1-S12 vs. C1-S22 | 1.21 |  | 0.99 | 1.00 | [0.67, 2.18] |
| Same-Trial Lure: C1-S12 vs. C2-S12 | 0.73 |  | -1.19 | 1.00 | [0.32, 1.67] |
| Other-Trial Lure: C1-S12 vs. C2-S12 | 0.86 |  | -0.27 | 1.00 | [0.15, 4.91] |
| Target: C1-S12 vs. C2-S12 | 1.45 |  | 1.79 | .88 | [0.76, 2.79] |
| Same-Trial Lure: C1-S22 vs. C2-S22 | 1.22 |  | 1.11 | 1.00 | [0.70, 2.12] |
| Other-Trial Lure: C1-S22 vs. C2-S22 | 1.01 |  | 0.02 | 1.00 | [0.21, 4.80] |
| Target: C1-S22 vs. C2-S22 | 1.24 |  | 1.50 | .98 | [0.80, 1.92] |
| Same-Trial Lure: C2-S12 vs. C2-S22 | 1.49 |  | 1.72 | .92 | [0.72, 3.06] |
| Other-Trial Lure: C2-S12 vs. C2-S22 | 0.67 |  | -0.75 | 1.00 | [0.12, 3.60] |
| Target: C2-S12 vs. C2-S22 | 1.02 |  | 0.15 | 1.00 | [0.61, 1.72] |
| Same-Trial Lure: C2-S22 vs. C2-S23 | 0.87 |  | -0.65 | 1.00 | [0.44, 1.71] |
| Other-Trial Lure: C2-S22 vs. C2-S23 | 0.73 |  | -0.47 | 1.00 | [0.09, 5.98] |
| Target: C2-S22 vs. C2-S23 | 1.10 |  | 0.62 | 1.00 | [0.67, 1.83] |
| ***RT***  Full model: AIC = 147124, BIC = 147270, *R^2^_adj_* = 0.588  Reduced model (both main effects): AIC = 147159, BIC = 147232, *R^2^_adj_* = 0.587  Reduced model (main effect probe type only): AIC = 147652, BIC = 147689, *R^2^_adj_* = 0.567  Reduced model (main effect selection demand only): AIC = 148146, BIC = 148205, *R^2^_adj_* = 0.548  Model comparison (full model vs. no interaction): *χ^2^(10)* = 55.50, *p* < .001  Model comparison (both main effects vs. probe type only): *χ^2^(5)* = 502.83, *p* < .001  Model comparison (both main effects vs. selection demand only): *χ^2^(2)* = 990.93, *p* < .001 | | | | | |
| Contrast | Estimated Mean Difference | *df* | *t*-value | *p*-value | 95% CI |
| Same-Trial Lure vs. Other-Trial Lure | 104 | 11004.1 | 19.46 | <.001 | [88, 121] |
| Target vs. Other-Trial Lure | -40 | 11004.0 | -8.65 | <.001 | [-54, -25] |
| Same-Trial Lure vs. Target | 144 | 11004.1 | 30.51 | <.001 | [129, 159] |
| C1-S12 vs. C1-S13 | -10 | 11004.0 | -1.26 | 1.00 | [-33, 14] |
| C1-S12 vs. C1-S22 | -74 | 11004.0 | -11.16 | <.001 | [-94, -53] |
| C1-S12 vs. C2-S12 | -43 | 11004.0 | -5.63 | <.001 | [-67, -19] |
| C1-S22 vs. C2-S12 | 31 | 11004.1 | 4.71 | <.001 | [10, 51] |
| C1-S22 vs. C2-S22 | -19 | 11004.0 | -3.49 | .013 | [-36, -2] |
| C2-S12 vs. C2-S22 | -50 | 11004.1 | -7.56 | <.001 | [-70, -29] |
| C2-S22 vs. C2-S23 | -31 | 11004.0 | -4.71 | <.001 | [-52, -10] |
| Same-Trial Lure: C1-S12 vs. C1-S13 | -25 | 11004.0 | -1.70 | .93 | [-70, 21] |
| Other-Trial Lure: C1-S12 vs. C1-S13 | -12 | 11004.0 | -0.89 | 1.00 | [-56, 31] |
| Target: C1-S12 vs. C1-S13 | 9 | 11004.0 | 0.86 | 1.00 | [-23, 41] |
| Same-Trial Lure: C1-S12 vs. C1-S22 | -102 | 11004.0 | -7.92 | <.001 | [-142, -62] |
| Other-Trial Lure: C1-S12 vs. C1-S22 | -46 | 11004.0 | -3.81 | .004 | [-85, -8] |
| Target: C1-S12 vs. C1-S22 | -73 | 11004.0 | -8.21 | <.001 | [-101, -45] |
| Same-Trial Lure: C1-S12 vs. C2-S12 | -85 | 11004.1 | -5.77 | <.001 | [-131, -39] |
| Other-Trial Lure: C1-S12 vs. C2-S12 | -40 | 11004.0 | -2.86 | .11 | [-84, 4] |
| Target: C1-S12 vs. C2-S12 | -3 | 11004.1 | -0.32 | 1.00 | [-35, 29] |
| Same-Trial Lure: C1-S22 vs. C2-S22 | -32 | 11004.0 | -3.03 | .067 | [-64, 1] |
| Other-Trial Lure: C1-S22 vs. C2-S22 | -9 | 11004.0 | -0.87 | 1.00 | [-40, 22] |
| Target: C1-S22 vs. C2-S22 | -16 | 11004.0 | -2.22 | .53 | [-39, 7] |
| Same-Trial Lure: C2-S12 vs. C2-S22 | -50 | 11004.1 | -3.85 | .003 | [-88, -9] |
| Other-Trial Lure: C2-S12 vs. C2-S22 | -15 | 11004.0 | -1.21 | 1.00 | [-53, 23] |
| Target: C2-S12 vs. C2-S22 | -86 | 11004.1 | -9.59 | <.001 | [-114, -58] |
| Same-Trial Lure: C2-S22 vs. C2-S23 | -18 | 11004.0 | -1.41 | .99 | [-58, 22] |
| Other-Trial Lure: C2-S22 vs. C2-S23 | -43 | 11004.0 | -3.58 | .010 | [-81, -6] |
| Target: C2-S22 vs. C2-S23 | -32 | 11004.1 | -3.52 | .012 | [-60, -4] |

*Note.* CI = confidence interval. *C1 = single context, C2 = dual context, S1 = single-target, S2 = dual-target*
